# Inhibition of the miR-1914-5p increases the oxidative metabolism in cellular model of steatosis by modulating the Sirt1-PGC-1α pathway and systemic cellular activity

**DOI:** 10.1371/journal.pone.0313185

**Published:** 2024-11-08

**Authors:** Thais Porto-Barbosa, Letícia Ferreira Ramos, Camila Cristiane Pansa, Letícia Ramos Molica, Osmar Malaspina, Karen C. M. Moraes

**Affiliations:** 1 Departamento de Biologia Geral e Aplicada, Instituto de Biociências, Universidade Estadual Paulista “Júlio de Mesquita Filho”—Campus Rio Claro, Rio Claro, SP, Brazil; 2 Instituto de Química, Universidade Estadual Paulista “Júlio de Mesquita Filho”–Graduate Program in Biotecnology, Campus Araraquara, Araraquara, SP, Brazil; 3 Instituto de Biociências, Universidade Estadual Paulista “Júlio de Mesquita Filho”–Graduate Program in Cellular, Molecular and Microbiology, Campus Rio Claro, Rio Claro SP, Brazil; Midwestern University, UNITED STATES OF AMERICA

## Abstract

Metabolic associated fatty liver disease (MAFLD) is considered an indicator of metabolic syndrome, which affects millions of people around the world and no effective treatment is currently available. MAFLD involves a wide spectrum of liver damage, that initiates from steatosis (fatty live) and may progress to more complex pathophysiology. Then, details in lipid metabolism controlling should be explored aiming to control the fatty liver. In this context, the miR-1914-5p can be considered a potential biotechnology tool to control lipid metabolism in hepatic cells. This miRNA finds potential mRNA binding sequences in more than 100 molecules correlated with energy production and lipid metabolism pointed in bioinformatic platforms. The present study addressed the miR-1914-5p effects in hepatic HepG2/LX-2 co-cultured cells in a *in vitro* steatotic environment stablished by the addition of 400 μM of a mixture of oleic and palmitic acids. The analyses demonstrated that the inhibition of the miRNA reduced energetic metabolites such as total lipids, triglycerides, cholesterol and even glucose. In addition, the miR-inhibitor-transfected cells did not present any deleterious effect in cellular environment by controlling reactive oxygen species production (ROS), mitochondrial membrane potential (ΔΨm) and even the pro-inflammatory environment. Moreover, the functional effect of the investigated miR, suggested its close connection to the modulation of Sirt-1-PGC1-α pathway, a master switch metabolic route that controlls cellular energetic metabolism. Our assays also suggested a synergistic effect of this miR-1914-5p in cell metabolism, which should be considered as a strong candidate to control steatotic environment in future clinical trials.

## Introduction

Metabolic associated fatty liver disease (MAFLD) is considered an indicator of metabolic syndrome, which affects millions of people around the world [1,2]. This pathology presents different etiologies and is responsible for several comorbidities that affects liver and extra-hepatic tissues [3,4]. Unfortunately, in its initial stages the disease causes no symptoms, and in the liver may progress to a more serious clinical condition, such as non-alcoholic steatohepatitis (MASH), fibrosis/ cirrhosis, or even hepatocarcinoma (HCC) [1,2]. In recent years, studies have investigated the mechanisms that support the establishment and progression of liver diseases in MAFLD; however, no effective therapeutic approaches are available, and they are fundamentally based on diet and lifestyle changes [2]. Histologically, the liver is constituted of parenchymal cells (80% of liver mass) and non-parenchymal cells (Kupffer cells, sinusoidal endothelial cells and hepatic stellate cells, HSCs) [5]. Physiologically, an initial marker of MAFLD is the presence of hepatic steatosis followed by some metabolic dysfunction. Steatosis or fatty liver is established when lipids accumulate in percentages equal to or above 5% of the liver weight [6,7]. In its early stage, the steatosis is characterized by increased deposition of triglycerides in liver cells as results from imbalanced mechanisms between the storage and the use of those molecules by cellular machinery. Fatty hepatocytes become more sensitive to oxidative stress and mitochondrial dysfunction, among others, which contributes to the toxicity of the cellular environment, disease progression and body dysfunction [8,9]. Thus, the control of fatty liver disease is a real concern worldwide.

To contribute to the field of fatty liver research many investigators use animal models. However, due to the international policy of reducing the use of animals in research, alternative methodological procedures have been adopted, and cell culture is considered an important tool in predictive toxicology research [10–13]. Cell culture methodology is reproducible if cautiously performed, which facilitates the investigation of cellular and molecular mechanisms in pathologies [14,15]. In the present study, we presented molecular details of the effects of a mixture of free fatty acids (FA) in co-culture of hepatocytes and hepatic stellate cells to simulate an *in vitro* steatotic environment. During steatosis, different cells are activated inside the liver, and along with hepatocytes the HSCs act increasing the pro-fibrosing elements [16] and disease progression to fibrosis and/or cirrhosis. HSCs represent 5–8% of all liver cells reinforcing its relevance on the establishment of MAFLD. Then, a co-culture model established using both cell lines, seeded together, allowed a more integrative study of cellular signaling and possibilities to better clarify mechanisms that supports the establishment and development of the hepatic fatty liver [17].

Moreover, using the same cellular model, we evaluated the molecular function of the miR-1914-5p in the investigated steatotic environment. Previous studies from our lab demonstrated that the microRNA (miRNA) 1914-5p plays a relevant function in the lipid metabolism in human hepatic cells [16,18]. Furthermore, bioinformatics analysis on free available platforms that explore miRNA interactions revealed several potential target sequences of the miR-1914-5p on more than 100 mRNAs that potentially plays relevant functions in control lipid metabolism and energy production. miRNAs are non-coding RNAs containing 21–25 nt that modulate gene expression by binding to the 3’-untranslated region (UTR) of the target mRNAs, reducing or blocking protein synthesis [19–21]. In the present study, using the co-culture cellular model of steatosis we investigated the effect of the miR-1914-5p in this deleterious environment. The results reinforced the functional activity of the investigated miR in lipid metabolism, as a promising molecule for future clinical investigation for liver lipid metabolic dysfunctions.

## Materials and methods

### Cell culture, *in vitro* steatotic model establishment, and cellular viability

The human HepG2 hepatocarcinoma cell (ATCC^®^ HB8065™) and the human HSC cell line, LX-2 [22] were maintained in low glucose Dulbecco’s Modified Eagle Medium (DMEM) supplemented with 10% fetal bovine serum (FBS) plus antibiotics under standard conditions (37°C, 5% CO_2_). The reagents were purchased from Thermo Fischer Scientific, USA. For co-culture experiments, HEpG2 and LX-2 cells (7:3) were seeded at 3 x 10^4^ cell/ cm^2^ in DMEM for all the analyses. The ratio (7:3) was selected based on De Oliveira Da Silva et al (2018) [16] reinforced by the fact that the HSCs represent 5–8% of all liver cells [23]. 96-well plates were used for cytotoxicity, ROS and ΔΨm assays; 24-well plates were used for fluorescent fat acid (FA) pulse-chase analyses; 6-well plates were used for triglycerides (TG) and cholesterol measurements, Fourier transform infrared spectroscopy (FT-IR) analyses, gas chromatography/ mass spectrometry (GC/MS) analyses of fatty acids, real time PCR, and dual-luciferase reporter assay; 75 cm^2^ culture flasks were used for western blot analyses. After the seeding, cells were maintained under regular culture conditions for a 48 h-interval, before their respective incubation or not with a 400 μM of a mixture of FA (containing oleic and palmitic acids (Merck-Milipore) at 2: 3 proportional rates, for an interval of 24 h at under standard culture conditions. To verify the effects of the vehicle in which FA was solubilized, cells were incubated with the equivalent volume (w/v) of dimethyl sulfoxide (DMSO). The maximum concentration of FA used in the *in vitro* steatosis induction was established through the classical MTT analyses [24], by the incubation of the cells with different amounts of a mixture of FA. For the MTT, the cultures were cultivated isolated or in co-culture, and after the treatments with the different concentrations of FA, the tetrazolium salt reagent (Merck-Milipore) was added to the cultures, and they were incubated for 4 h at 37°C. The resulting formazan crystals were dissolved with DMSO, and cell viability was evaluated at 570 nm in Infinite M200 Pro™ Microplate Reader (Tecan, Germany).

The assays were repeated at least three times and cells were free of contamination. All the experimentation procedures followed UKCCCR Guidelines for the Use of Cell Lines in Cancer Research.

### Cellular transfections for functional assays

For miRNA transfections, HepG2 and LX-2 co-cultures were also seeded as described above in a 24-well plate. After the seeding, cells were maintained under regular culture conditions for 24 h and next transfected with 40 nM of miR-1914-5p mimic or inhibitor or non-targeting miRNAs controls (mirVana™miRNAs, Thermo Fisher Scientific, USA) [16,18]. After 24 h-transfection, to the cells were added 400 μM of a mixture of FA containing oleic and palmitic acid (2: 3 proportional rates) and incubated again for another 24 h under standard culture conditions (37°C, 5% CO_2_). Next, cells were collected for further investigation.

To validate the miR transfection protocol efficiency in the hepatic co-culture, they were transfected as above described, using 40 nM BLOCK-iT™ Alexa Fluor Red Fluorescence Control (Thermo Fisher Scientific). Next, cells were fixed in paraformaldehyde at 1.4%, the nuclei were stained using 4′,6-Diamidino-2-phenylindole dihydrochloride (DAPI, Merse, German) and cells were analyzed under fluorescent microscopy, according to Silva et al. (2020) [25]. The analyses were performed at least In triplicates, and the results were plotted in graphs using Graph Pad Prism^®^ 8.

### Triglycerides and cholesterol measurements

Triglycerides and cholesterol were measured using Triglycerides Liquiform and Cholesterol Liquiform (Labtest, Brazil) reagents, adapting manufacturer’s instructions. For the analyses, co-culture of HepG2 and LX-2 (7:3) cells were cultivated in 6-well plates as described above and next they were transfected or not with mirVana™ miRNA (Scramble miRNA used as negative control). After 24 h, to the cells were added or not 400 μM of a mixture of FA. Cells were incubated again for another 24 h and, next, they were collected and suspended in 100 μL of phosphate-buffered saline (PBS), containing or not 0.68 mM EDTA, and used in TG and cholesterol measurement in a BioMate™ 3 Series spectrophotometer (Thermo Fisher Scientific, USA). The analyses were performed at least three times, and the results were plotted in graphs using Graph Pad Prism^®^ 8.

### Fourier transform infrared spectroscopy analysis

FTIR spectroscopy spectra were obtained using universal attenuated total reflectance (UART-FITR). For the assays, co-culture of HepG2 and LX-2 (7:3) cells were cultivated in 6-well plates as described above and they were, submitted to different treatments as described previously (controls, FA, and mirVana™ molecules), collected and dried. The samples were analyzed in a Nicolet10 FT-IR Spectrometer (Thermo Fisher Scientific, USA) and the spectra were recorded between the regions 4000 cm^-1^ and 450 cm^-1^ at 20°C. Thirty-two scans were obtained with resolution of 4 cm^-1^ and data were processed with Spectrum 5.2 software (PerkinElmer, USA). The spectra were normalized to amide I band (and A1651) and the baseline-corrected spectrum was considered to determine the variations. The protocol was adapted from [26,27]. The analyses were performed at least In triplicates, and the results were plotted in graphs using Graph Pad Prism^®^ 8.

### Gas chromatography/ mass spectrometry (GC/MS) analyses of fatty acids

For the GC/MS analyses, co-culture of HepG2 and LX-2 (7:3) cells were cultivated in 6-well plates as described above and submitted to different treatments as described previously). Next, cells were collected, and the fatty acids were extracted following the FA esterification methodology described by Fischer and Speier (1895) [28]. The FAs were measured in a GCMS-QP2010 Plus machine (Shimadzu Scientific Instruments, Japan) with an RTX MS capillary column (30 m × 0.25 mm ID, 0.25-μm thick film) coupled with a mass-selective detector (Shimadzu GCMSQP2010 Plus). Standard curves for FAs quantification were obtained using dilutions of palmitic, and oleic acids (Sigma-Aldrich, USA). The analyses were performed at least In triplicates, and the results were plotted in graphs using Graph Pad Prism^®^ 8.

### Intracellular reactive oxygen species (ROS) and mitochondrial membrane potential (ΔΨm)

For ROS and ΔΨm measurements, co-culture of HepG2 and LX-2 (7:3) cells were cultivated in 96-well plates as described above. ROS was measured with the Fluorometric Intracellular ROS Kit (Merck–Sigma Aldrich), following the supplier’s instruction. The fluorescence intensity was evaluated at 675 nm. The ΔΨm was assessed with the Mitochondrial Membrane potential Kit (Merck–Sigma Aldrich), also following the supplier’s instruction. The fluorescence intensity was evaluated at 540 nm. Both analyses were performed in Infinite M200 Pro™ Microplate Reader (Tecan, Germany). The results were repeated at least In triplicates, and the results were plotted considering arbitrary unit of fluorescence in graphs using Graph Pad Prism^®^ 8.

### Bioinformatic analyses of potential mRNA targets for miR-1914-5p

Public online databases were used aiming to identify potential mRNA targets for miR-1914-5p as detailed in Shaker et al., (2020) [29]. Information described at miRbase (http://www.mirbase.org), TargetScan (http://www.targetscan.org), miRTar (http://miRTar.mbc.nctu.edu.tw), miRDB http://mirdb.org and microRNA.org (http://www.microrna.org) were used to address those potential target sites on mRNAs. The search focused on the identification of mRNAs correlated with the energetic and lipid metabolism, that potential interacts with the miR-1914-5p. The functional activity of each mRNA was confirmed on Gene Cards (https://www.genecards.org) and they are free available.

### Real time PCR (qRT-PCR)

Total RNA and miRNA were extracted from co-culture of HepG2 and LX-2 (7:3) cells were cultivated in 6-well plates as described above and submitted to all the investigated conditions, The nucleic acids were isolated, reversed transcribed and amplified according to De Oliveira Da Silva et al (2018) [16]. S1 Table presents specific primers sets used and designed using Primer3Plus platform (http://www.bioinformatics.nl/cgi-bin/primer3plus/primer3plus.cgi). The mRNA and miRNAs expression analysis were performed in triplicates and normalized to the reference gene *β-ACTIN* or U6, respectively. The 2-^ΔΔCT^ method was used to relative quantification and graphs were plotted using Graph Pad Prism^®^ 8. The analyses were performed at least In triplicates.

### Fluorescent FA pulse-chase

Fluorescent microscopy for fatty acid tracking inside the cells were performed adapting in Silva et al. (2020) and Rambold et al., 2015 [25,30]. For that, co-culture of HepG2 and LX-2 (7:3) cells were cultivated on 13 mm circular coverslips in 24-well plates as described above and submitted to all the investigated conditions, After seeding, cells were transfected or not with different mirVana™miRNAs and incubated for 24 h at standard culture conditions, and, then, 400 μM of a mixture of FA were added or not to each independent group of cells. Next, 1 μM of BODIPY™ 558/568 C_12_ (Bodipy-C_12_) (4,4-Difluoro-5-(2-Thienyl)-4-Bora-3a,4a-Diaza-s-Indacene-3-Dodecanoic Acid) (Thermo Fisher Scientific, USA) were added to the cultures. Those cells were cultivated for 16 hours under regular conditions, and next, the culture medium containing the Bodipy-C_12_ was replaced by a fresh one without C_12_. After 1 h incubation in this fresh medium, group of cells were fixed using paraformaldehyde solution or further incubated for 8 hours before their fixation. The control assays were performed incubating the cells with 50 μM of etomoxir sodium salt (Merck, Germany). The analyses were performed at least In triplicates.

To complete the analyses, LDs were stained with 0,5 μg/mL of BODIPY™ 493/503 (4,4-Difluoro-1,3,5,7,8-Pentamethyl-4-Bora-3a,4a-Diaza-sIndacene) (Thermo Fisher Scientific, USA) for 20 minutes, followed by successive washes in phosphate buffer solution (PBS), and cell nuclei were stained with DAPI. The images were taken using an Olympus BX51 microscope and a DP71 digital photographic system and they were quantified using the Image-J software (http://rsbweb.nih.gov) with the "JACoP" plug-in to obtain a Pearson’s coefficient between Bodipy-C_12_ overlap and lipid droplets. The analyses were performed at least In triplicates, and the results were plotted in graphs using Graph Pad Prism^®^ 8.

### Western blot

Western blot assays were performed, visualized, and analyzed according to Da Silva et al., (2016) [31]. For that, membranes containing protein from different cellular groups were overnight incubated with anti- Peroxisome Proliferator-Activated Receptor Gamma Coactivator 1α (PGC-1α, Sigma-Aldrich, USA) or anti-Sirt1 (D1D7) (Cell Signaling, USA) monoclonal antibodies, and anti-FOXO-1 (α-FOXO1A, FineTest, China), or Acetyl-FOXO1 (Lys 294) antibody or anti-β actin (Cell Signaling, USA) polyclonal antibodies, followed by 2 h of incubation with a horseradish peroxidase-conjugated secondary antibodies (Cayman Chemical, USA). Immunoreactive bands were visualized using the chemiluminescent detection kit (ECL™, GE Healthcare, USA) and the C-Digit Blot SCanner (Li-Cor). The corresponding protein bands were quantified using the software Image-J (http://rsbweb.nih.gov). The analyses were performed at least In triplicates, and the results were plotted in graphs using Graph Pad Prism^®^ 8.

### Construction of recombinant mammalian expression systems

Total RNA from HepG2 were isolated, reversed transcribed and specific gene sequences were amplified according to regular protocols in molecular biology. The full-length cDNA corresponding to the open reading frame from the gene PGC-1α (AF106698.1) and Sirt1 (NM_012238.5) were inserted in the polylinker cloning site of pcDNA3.1 (+) according to the gene specific sequence requirements. Next, recombinant plasmids or empty plasmids were used to transform *Escherichia coli* DH5a. Clones were selected with ampicillin and the pcDNA3.1 constructs were purified from the bacteria. The recombinant plasmids were sequenced and next used for cellular transfection of mammalian cells using regular protocol described in our lab [16,18]. Cellular clones were selected using neomycin. HepG2 and LX-2 were independently transfected. After the selection of stable clones, they were co-cultured and used to validate the previous results.

### Dual-luciferase reporter assay

Approximately 200 pb sequences from the 3’-UTR sequences of acetyl-CoA carboxylase (ACC2), carbohydrate response element bindidng protein (ChREBP), PGC-1α, peroxisome proliferator-activated receptor (PPAR)-γ, and Sirt1 containing the putative binding site for the miR-1914-5p (seed sequence), or a mutated seed sequence according to miRbase were amplified by PCR and cloned in the pGL3-Control vector (Promega, USA). These vectors were co-transfected with miR-1914-5p mimic or inhibitor into the LX-2 cells using Lipofectamine^®^ RNAiMAX Transfection Reagent (Thermo Fisher Scientific, USA) adapting Silva et al., (2016) [31]. The Renilla luciferase reporter plasmid (pRL-TK) was used as the internal control for transfection efficiency. The assays were measured in a TD20/20 luminometer (Turner Designs). The analyses were performed at least In triplicates, and the results were plotted in graphs using Graph Pad Prism^®^ 8.

### Graphs and statistical analyses

Multiple data are presented as mean ± standard derivations (SD) from at least 3 independent experiments. Graphs and statistical analyses were generated using Graph Pad Prism^®^ 8 software. The differences between the cellular groups were calculated using one-way analysis of variance (ANOVA), followed by Dunnett’s test. Significance was set at *p < 0.05.

## Results

### Assessing cellular viability

**Fig 1:**
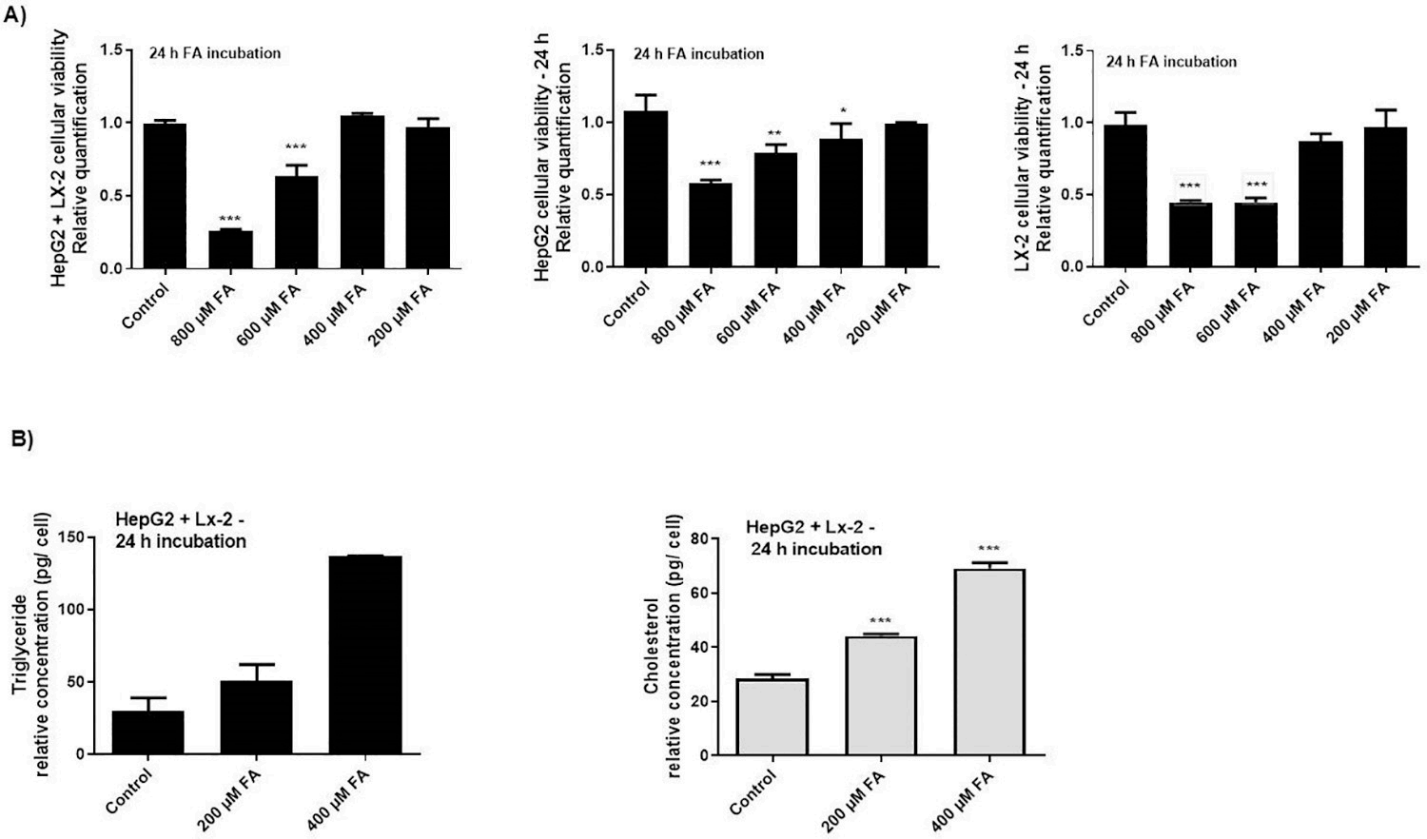
Establishing *in vitro* steatotic cellular model. A) HepG2 and LX-2 cells were seeded at the proportion rate of 7:3 units and cultivated for 24 h under regular conditions. Next, different concentrations of FA mixture were added to the cultures to simulate *in vitro* steatotic condition. After 24 h cell viability was investigated by MTT analyses; B) Triglycerides and cholesterol levels measurement after FA were added to the cellular co-culture mode. The graphs represent the mean values of at least three independent experiments (*p<0.05).

The exposure of hepatic cell lines (HepG2, or LX-2, or the co-culture of both of them (7:3) to culture media containing different concentrations of a mixture of fatty acid (FA) (2:1 oleic: palmitic acid) for 24 h-incubation resulted in viability rates lower than 80%, when cells were exposed to the concentrations of 800 μM and 600 μM, compared to viable cells at the control condition (Fig 1A). The concentrations of 400 μM and 200 μM of FA were initially considered for further analysis because they slightly affected cellular viability in all investigated groups of cells. These analyses were required to assure that all the further analyses, presented in this study, were conducted with viable cells, specially because each cell line present metabolic particularities. In addition, to verify the effect of the vehicle in which FA was solubilized, cells were incubated with the equivalent volume (w/v) of dimethyl sulfoxide (DMSO) of each FA concentration (S1A Fig). At the concentrations of 400 μM and 200 μM of FA, the equivalent volume of DMSO did not reduce the viability of the cultures.

Next, to verify the effect of the 400 μM and 200 μM of FA in hepatic cells for 24 h-incubation, triglycerides and cholesterol levels were accessed (Figs 1B and S1B). The results demonstrated that the addition of 400 μM of FA in cell culture accumulated the highest amount of those metabolites, simulating a better *in vitro* steatotic environment in co-cultures. The effect of FA in LX-2 and HepG2 were also verified (S1C Fig) and the 400 μM of FA also induced more changes in the basic energetic metabolites evaluated. After these initial analyses, the next following assays were performed with then hepatic co-culture of HepG2 and LX-2 (7:3) cells, considering the interconnected metabolism between different cell lines [17], which is more representative of basic cellular communication that happens in an entire organ.

### Modulatory effects of miRNA-1914-5p on cellular steatotic environment

**Fig 2:**
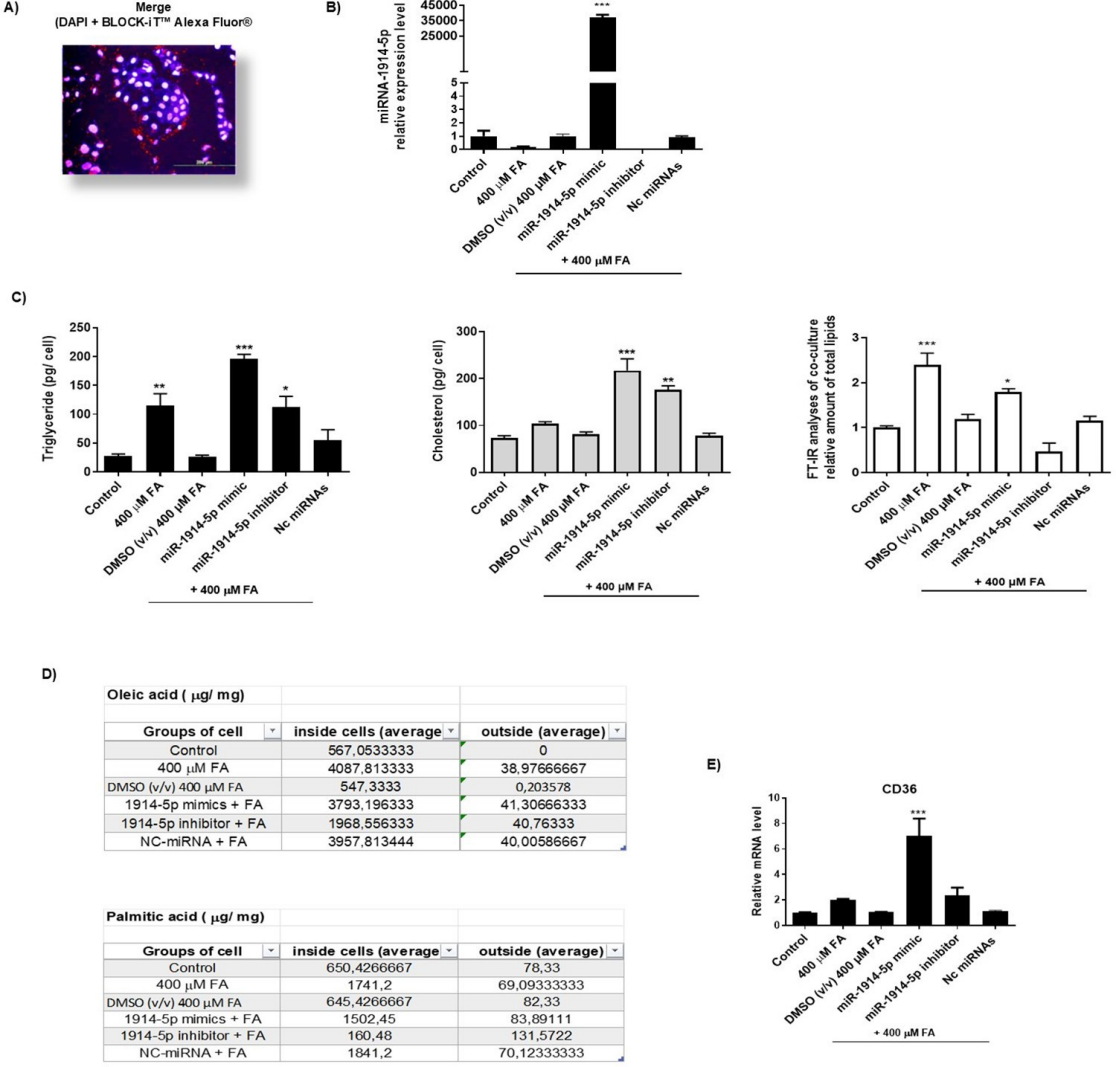
Molecular and biochemical effects of miR-1914-5p on lipid metabolism. A) microscopy analyses of hepatic co-cultures transfected with the miRNA-1914-5p; (B) qRT-PCR of miRNA expression levels in different group of cells. C) Biochemical measurements of triglycerides, cholesterol and total lipids in different groups of cells. D) Gas chromatography/ mass spectrometry (GC/MS) analyses of fatty acids in investigated group of cells. E) qRT-PCR of transcript levels of CD36. The graphs and images present mean values of the average from at least three independent experiments. ANOVA testing showed significant differences (*p< 0.05) in the assays. Nc-miRNA = negative control (Scramble miRNAs).

To validate the efficiency of the transfection methodology, the co-cultures were transfected with fluorescent miRNA control BLOCK-iT™ Alexa Fluor Red Fluorescence Control (Thermo Fisher Scientific). The results demonstrated that the methodology was efficient for the co-culture transfection (Fig 2A). Then, after cells treatments the miRNA-1914-5p level was quantified by, qRT-PCR (Fig 2B). Compared to the levels found in control group, in cells exclusively incubated with 400 μM of FA the relative expression levels of the investigated miR reduced; in cells incubated with the FA-solvent (DMSO), the miR expression pattern did not change, compared to the untransfected and FA-treated groups (control condition). In transfected cells, the investigated miRNA extremely increased when cells were transfected with the miR-1914-5p mimics molecule; null amount of this miRNA was observed in miR-1914-5p-inhibitor transfected cells. In addition, cells transfected mirVana™miRNA (negative control) did not change the expression levels of the miR-1914-5p, when compared to the untransfected and FA-treated groups.

Next, we functionally validated the effect of the miR-1914-5p in metabolites syntheses correlated with the steatosis in the established cellular model (Fig 2C). In cells exclusively incubated with 400 μM of FA, TG levels increased ~4 x, cholesterol increased ~3X, and total lipids increased– 2 x, compared to the levels found in the control group of cells.

When cells were transfected with the mimic molecule, before the FA addition to the culture, all the investigated metabolites increased exponentially. However, in the miR-inhibitor-transfected cells, TG levels were the same as found in the FA-group of cells; cholesterol reduced—42% compared to the level found in FA-treated cells. In addition, the total lipids were extremely reduced by ~5 x in miR-inhibitor-transfects cells, compared to the other groups. In DMSO-treated cells no relevant changes were observed for the investigated molecules, when compared to the results found in the control group; otherwise, in cells transfected with the mirVana™miRNAs negative control. the results presented similar pattern to observed in FA-treated cells.

In addition, GC/MS analyses were performed to better clarify the effects of the mixture of FA composition added to the cell culture in lipid metabolism. Fig 2D presents the results of oleic and palmitic acids found inside and outside the cells for each investigated group. The analyses revealed higher levels of oleic acid inside the cells, compared to levels found outside the cells in culture media. In control group of cells, oleic acid was not found in the culture media; similar results were observed in DMSO-treated cells. Particularly, in transfected cells the presence of the miR-inhibitor reduced by ~ 51.8% the amount of oleic acid inside the cells, when compared to the untransfected group of cells incubated with 400 μM of FA, suggesting the activation of cellular metabolism that consumes or blocks this FA accumulation. The same GC/MS analyses for the quantification of palmitic acid inside and outside the cells also revealed higher amount of palmitic acid inside the cells. In miR-inhibitor-transfected cells palmitic acid was found reduced by 90.78% compared to the levels found in the untransfected group of cells incubated with 400 μM of FA. In addition, in miR-inhibitor-transfected cells, the amount of palmitic acid found outside those cells were ~90.48% higher than those found in FA group of cells. The results combined suggested that the miR-inhibitor-transfected cells direct the cellular metabolism to decrease the amount of palmitic acid inside the cells, which is well known to be toxic for the cells [32]. The same analyses were performed for cells incubated with DMSO, which present no relevant changes in percentages of oleic or palmitic acid inside and outside the cells, compared to the levels found in control cultures. For mirVana™miRNA negative controls, the observed results were like the one found in the untransfected and FA-treated cells.

To verify whether the above metabolic changes may correlate with changes in *CD36* gene expression, q-RT-PCR was performed for all groups of cells. CD36 is a protein located at the cell membrane that transports fatty acids collaborating with the lipid metabolism [33]. The results demonstrated that only in cells transfected with the miR-mimic the CD36 transcripts increased expressively; 7 x higher levels were found in this group of cells compared to the levels seen in the control group (Fig 2E). In DMSO-treated cells, no relevant changes in gene expression were observed, compared to the control levels. For negative control group of transfections, the results presented similar pattern to observed in FA-treated cells. Those results combined reinforced the suggestion that the presence of miR-mimics changes cellular machinery to accumulate fatty acid.

Considering all these changes in cellular metabolism mediated by the miRNA, the presence of a pro-inflammatory environment in the co-culture cellular model was investigated, by measuring transcription levels of annexin 1 (a molecule that is correlated with the control of the pro-inflammatory environment) and arachidonic acid (AA) using GC/MS. The results presented in S2 Fig demonstrated significant increase in annexin 1 expression and reduced synthesis of AA in mir-inhibitor-transfected conditions. In DMSO-treated cells no relevant changes were observed for the investigated molecules, when compared to the results found in the control group; otherwise, in cells transfected with the negative control. the results presented similar pattern to observed in FA-treated cells.

Next, ROS production and ΔΨm were measured for all investigated group of cells (S3A Fig). Considering ROS measurements, increased levels of this metabolites were observed only in the group of cells exclusively incubated with the FA. For ΔΨm, no significant change was observed in different groups of cells, when comparing to the control group (S3B Fig). In DMSO-treated cells no relevant changes were observed for the investigated molecules, when compared to the results found in the control group; otherwise, in cells transfected with the negative control. the results presented similar pattern to observed in FA-treated cells.

The results combined reinforced the use of the established cellular model as an alternative approach for hepatic liver disease, and the modulatory effects of the inhibition of miR-1914-5p to help control levels of fatty acids in cells, without disrupting homeostasis.

### Assessing metabolic routes modulated by miRNA-1914-5p on the *in vitro* steatotic cellular environment

**Fig 3:**
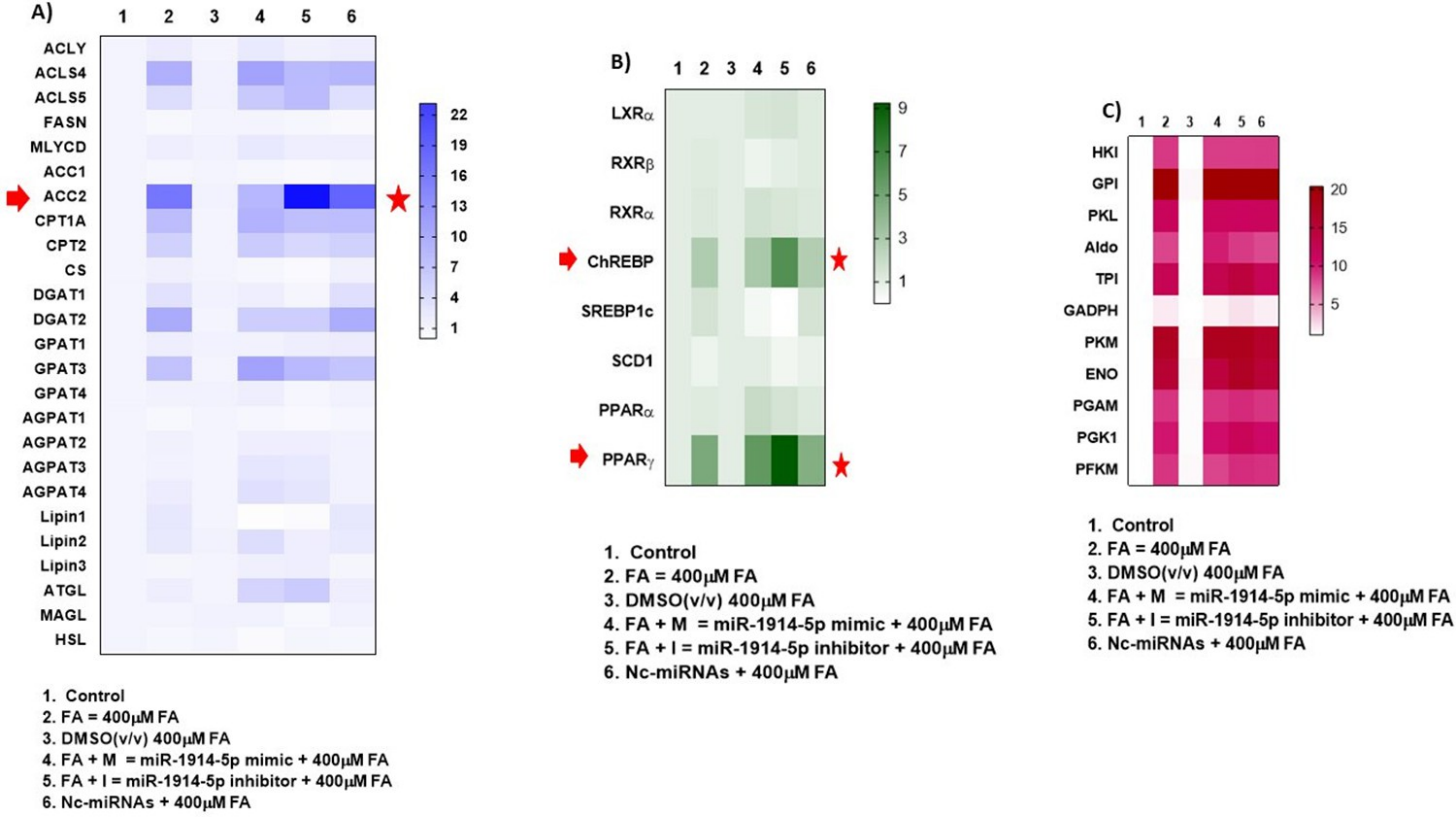
Heatmaps of the mRNA expressed between investigated cellular groups in q-RT-PCR analyses. Each column represents an individual cellular group, and each row represents an individual mRNA. Colors of the heatmap represent the Z-score: Higher–blue/ green/red, lower–white. The heatmap was packed using Graph Pad Prism^®^ 8 software. ANOVA testing showed significant differences between the cellular groups, and significance was set at *p<0.05. The Figure represents mRNAs found in A) lipid biosynthesis and packing; B) transcription factors and central molecules on bioenergetic routes; C) glycolytic enzymes. Legends (C = control; FA = fatty acid; M = miR-1914-5p mimics; I = miR-1914-5p inhibitor; Nc-miRNA = negative control, Scramble miRNAs, mirVana™).

To investigate the functional activity of the miR-1914-5p in the *in vitro* steatotic cellular model, the expression pattern of the key genes correlate with energetic metabolism were evaluated. The selected genes for qRT-PCR analyses were chosen based on their relevance in the investigated metabolism, despite the presence or not of potential mRNA target sites for the miR-1914-5p. The main goals were to investigate the effects of the miR in the expression pattern of relevant molecules also considering its indirect effects on the energetic metabolism. miRNAs may modulate the activity of transcriptional regulators and cofactors elements through RNA interference (RNAi), which collaborate with gene expression, and, consequently, the activation and/ or repression of molecular routes systemically. Fig 3 presents heatmaps from the analyses of gene expression. Fig 3A presents the results for genes correlated with the FA synthesis and an expressive increase in the levels of ACC2 was observed in cells transfected with the miR-inhibitor (~23-fold increase). In cellular groups exclusively incubated with the 400 μM of FA, ~6-fold increase was observed in gene expression compared to the levels found in control groups of cells). In DMSO-treated cells no relevant changes were observed for the investigated molecules, when compared to the results found in the control group. Otherwise, in cells transfected with the negative control. the results presented similar pattern to observed in FA-treated cells. Considering ACC2, the corresponding protein is associated with the mitochondria [34] and irreversibly catalyzes the carboxylation of acetyl-CoA to malonyl-CoA, the rate-limiting step in fatty acid synthesis [35]. Higher expression of ACC2 in the 1914-5p miR-inhibitor group suggested modulatory effects in β-oxidation [36,37].

In addition, the analyses of relevant molecules on bioenergetic routes (Fig 3B) point to the effects of the miR-inhibitor on increasing the expression levels of ChREBP (6.1-fold increase) and PPARγ (9.2-fold increase), comparing to the levels found in the control group of the assays (the untreated and untransfected cells). In FA-cellular groups, the levels of those genes also increased 9.8-fold and 9.2-fold, respectively, when compared to the control levels. No relevant changes were observed for DMSO and in negative control group, compared their results to observed in their control groups. ChREBP and PPARγ are relevant and central molecules in the energetic metabolism [38,39]. ChREBP plays relevant function in the *de novo* lipogeneses in liver and adipose tissues, and also works in a synchronized manner regulating the carbohydrate metabolism to maintain the body’s homeostasis [40,41]. PPARγ regulates fatty acid storage and carbohydrate metabolism [42] and has also been implicated in several diseases including obesity and diabetes [43]. The increased expression of both genes suggested that cells is trying to compensate for metabolic changes verified in lipid metabolism in transfected cells to stay alive. To clarify the effects of the miR-1914-5p in glucose metabolism in cells, gene expression from glycolytic enzymes was measured. Fig 3C presents the results, and the presence of the FA in group of the cells (transfected or untransfected) statistically increased the gene expression levels for all the investigated molecules, compared to the control group. Otherwise, the DMSO-treated cells did not change the expression level of the evaluated genes compared to the control group of cells. Together, the results reinforced an adaptive metabolism of cells either to the presence of the FA or the miRNAs.

### Cellular effects of miR-1914-5p in lipid oxidation of hepatic co-culture

To evaluate the modulatory effects of the miR-1914-5p in lipid oxidation in our steatotic cellular model, we focused on qRT-PCR results of carnitine palmitoyltransferase (CPT)1, CPT2, and citrate synthase (CS) (Fig 3A). CPT1 localizes at the cytoplasmic face of the mitochondria membrane and helps control mitochondrial β-oxidation [35], because it regulates acyl transport into the organelle. Considering all the investigated conditions, CPT1 levels increased considerably in all conditions where FA was added (~6.5-fold increase in average for all the stimulated conditions), compared to the control condition of the assay. However, CPT1 levels were reduced in cells transfected with miR- inhibitor, compared to the levels found in cells transfected with the miR-mimic (*p<0.05). The results for CPT2 expression were like CPT1. The protein CPT2 localizes in the mitochondrial inner membrane and acts synchronously with CPT1 to transport acyl groups into the organelle, controlling lipids oxidation [35,44]. Increased levels of CPT2 transcripts were observed in all conditions where FA was added (~4.3-fold increase in average for all the stimulated conditions), compared to the control condition of the assay. CPT2 levels were reduced in cells transfected with miR-inhibitor, compared to the levels found in cells transfected with the miR-mimic (***p<0.0001). Next, CS gene expression was evaluated. CS correlates with mitochondrial oxidative metabolism and is the first enzyme of the citric acid cycle [35,45]. In our assays, the presence of miRNA mimic or inhibitor reduced the expression levels of this enzyme compared to the control group in the assay or the FA-incubated cells. In the miR-inhibitor-transfected cells, the expression levels of CS were even lower (***p<0.0001), than the other investigated conditions. The results reinforced the existence of activated cellular mechanisms, unique for each investigated condition, to maintain the cell alive.

**Fig 4:**
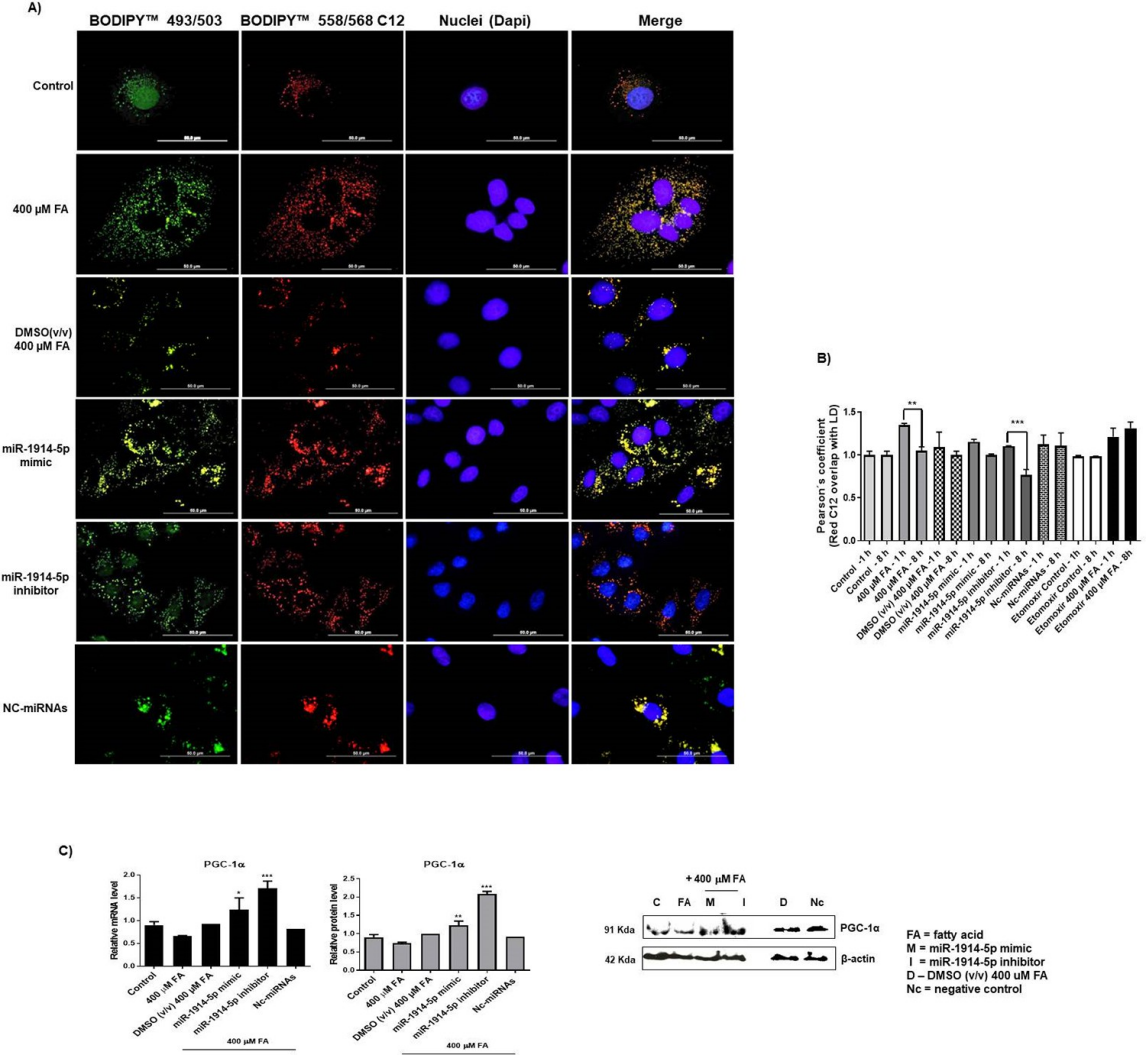
Effects of the miRNA-1914-5p mimics or inhibitor in co-culture of hepatic cells cultivated or not in the presence of a mixture of FA. A) Fluorescent FA pulse-chase. The images present BODIPY 558/568 C_12_ and the overlap between the lipid droplets and the cell nucleus for different treatments. B) The quantification of LD was calculated and is presented in the graph representing Person’s coefficient. C) mRNA and protein levels of PGC1-α in different groups of cells. Graphs represent the mean ± SD from at least three independent experiments and the statistics was performed using one-way analysis of variance (ANOVA), followed by Tukey’s test, between the investigated groups. The significance level at the statistical analyses was set at p<0.01 (*).

Additionally, the effects of the miR-1914-5p were evaluated by the fluorescent pulse-chase method to determine the β-oxidation of FA, through the quantification of the labeled lipids inside the cells (Fig 4A and 4B). The analyses were based on microscopic investigation. The absorbed lipids Bodipy-C_12_ were stored in cellular droplets and then quantified. Thus, the analyses were processed by measuring the Bodipy-C_12_ in LDs over time, and Pearson’s coefficients were calculated. A coefficient closer to one indicates high overlap between the images, demonstrating that Bodipy-C_12_ was stored in droplet form [25,30]. Cells exposed to 50 μM of etomoxir were considered the experimental control, because the drug inhibits CPT-1 enzyme. Fig 4A and 4B present the results. Using the two-time points microscopy analyses (1 h and 8 h after removal of the culture medium containing Bodipy-C_12_), the overlapping images illustrated the pattern of LD metabolization for each investigated condition. The groups of cells incubated with etomoxir, accumulated the intracellular lipids over the time-points, and presented a significant increase in the Pearson’s coefficient (***p<0.0001), compared to the control, indicating low rates of oxidative metabolism, as expected. Considering the investigated groups of cells, their metabolism along the 8 h time-point after removal of C12, revealed that the group of cells incubated exclusively with the 400 μM FA consumed the lipid (~20% reduction, compared to the C12 levels found at the 1 h time-point). In the cells transfected with the miR-mimics a ~14% reduction in C12 levels was found, and in the miR-inhibitor transfected cells displayed a 31% reduction in C12, suggesting the presence of effective oxidative processes in those groups of cells (***p<0.0001). The analyses for the DMSO-treated cells and for the cells transfected with the negative control did not present relevant changes, comparing the obtained results to the control group (untreated and untransfected cells).

To add more details on oxidative mechanisms, the transcript and protein levels of the peroxisome proliferator-activated receptor coactivator-1α (PGC)-1α were evaluated. PGC-1α acts as a regulator of lipid metabolism and oxidative processes by activating central genes in those processes [46]; furthermore, it helps control the production of ROS and the inflammatory processes in an interconnected manner [47]. The results presented in Fig 4C revealed increased expression for PGC-1α mRNA and protein levels (~40% increase) in miR-mimic transfected cells, compared with the untransfected and untreated groups of cells (control group). In miRNA-inhibitor-transfected cells, PGC-1α mRNA increased by ~96% and proteins levels increased ~ 138%. This higher expression of PGC-1α in cells transfected with the miRNAhibitor, supportteds many of the results observed in those cells, considering the activation of lipid expenditure and the observed decreased in ROS and AA productions (S2 and S3 Figs). The results combined reinforced the activation of interconnected signaling pathways to maintain cells alive.

**Fig 5:**
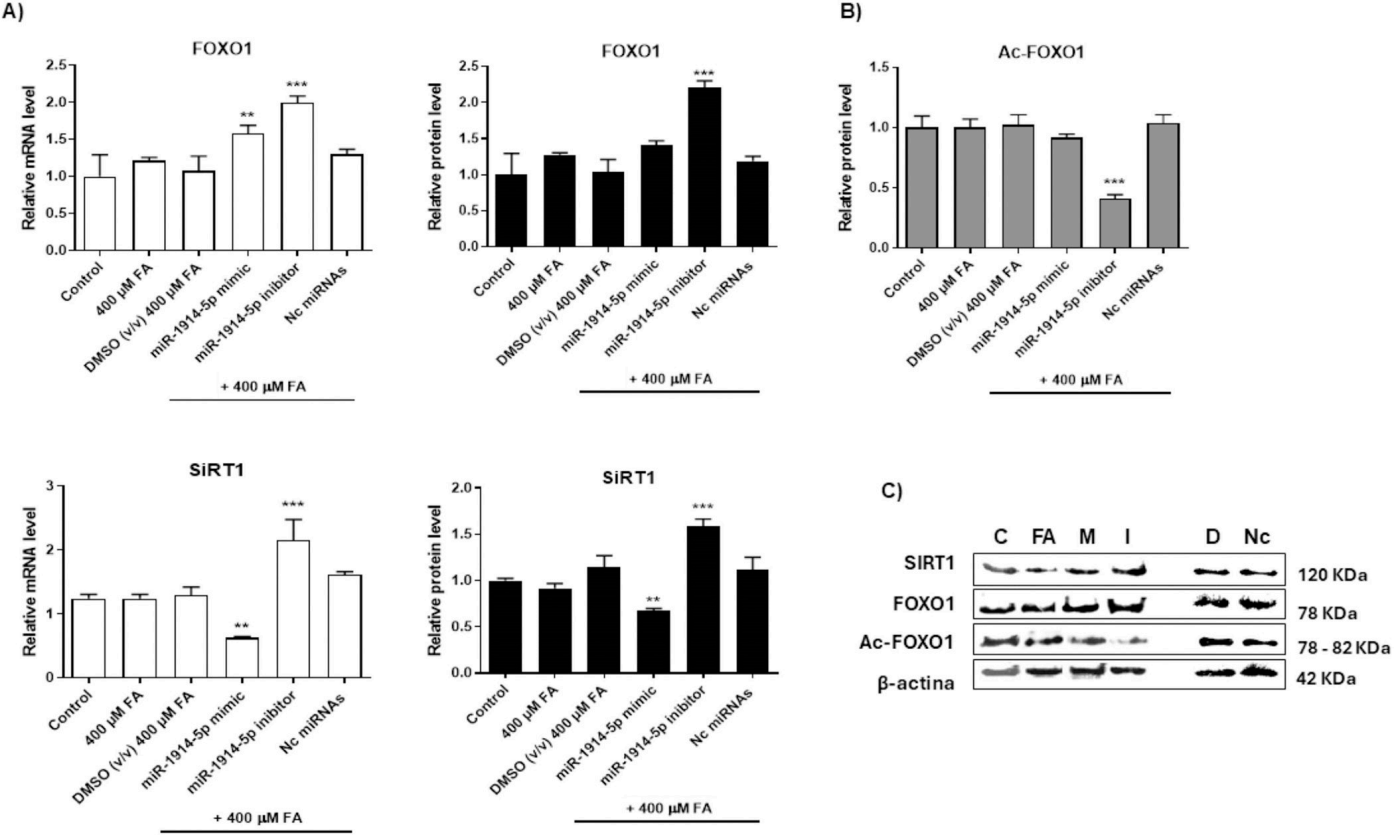
Functional activities of miR-1914-5p in relevant elements that control bioenergetic metabolism in co-culture of hepatic cells. A) Sirt1 and FOXO1 mRNA and protein levels in different groups of hepatic co-cultivated cells. B) In addition, the acetylated FOXO1 protein was measured. C) Representative western blotting from investigated cellular groups; β-actin was used as the loading control. The graphs and images present mean values of the average from at least three independent experiments. ANOVA testing showed significant differences (*p< 0.05) in the assays.

Among the cellular elements that regulate PGC-1α, the transcription factor forkhead box class-O1 (FOXO1) is appointed [48]. PGC-1α and FOXO1 work as metabolism sensors regulating several molecular routes. Among them, FOXO1 responds to insulin, regulating glucose levels and gluconeogenesis at cellular levels, which contributes to cellular homeostasis and viability [49]. In our model, despite the increased levels of FOXO1 transcripts and protein in cells transfected with the miRNA inhibitor (Fig 5), the glucose levels and some important genes stimulated by FOXO1 as glucose-6-phosphatase (G6Pase) and phosphoenolpyruvate carboxykinase (PEPCK), maintained low levels in miRNA-inhibitor-transfected cells, when comparing to the levels found in control cultures (S4 Fig). In our assay, both FOXO1 transcripts and protein levels presented similar expression patterns in all the investigated cellular groups. A ~ 2-fold increase in FOXO1 levels were observed in cells transfected with the miR-inhibitor molecule. In addition, the acetylated levels of FOXO1 protein were evaluated using Western Blotting. In the miR-inhibitor-transfected group of cells, ~60% reduction in FOXO1 acetylation levels were observed, resulting in increased amount of deacetylated protein. It is well described that the deacetylation of FOXO1 protein protects cells against their own destruction, because it limits mitochondrial lipid utilization through FA oxidation [50]. Moreover, increased deacetylation levels induce changes in the half-life of FOXO1 transcripts, resulting in a dynamic synthesis and degradation of the molecule [51], which supports the high mRNA levels of FOXO1 in the miR-inhibitor-transfected group of cells. In addition, increased levels of deacetylated FOXO1 retain the protein in the nucleus, enhancing its transcriptional activity that promotes the expression of several genes.

To verify if Sirt1 modulates FOXO1 deacetylation in our cellular model, mRNA and protein levels of Sirt1 were investigated (Fig 5). The analyses demonstrated that those molecules presented similar patterns of expression in all the investigated sample. In addition, in miR-inhibitor-transfected cells, Sirt1 presented more pronounced increase (~2.15-fold and 1.6-fold for RNA and protein levels, respectively). These results suggested an interconnect modulatory effect between Sirt1 and FOXO1 activity in the miR-inhibitor-transfected cells, which co-regulates PGC-1α levels [52]. In DMSO-treated cells no relevant changes were observed for the investigated molecules, when compared to the results found in the control group; otherwise, in cells transfected with the mirVana™miRNAs negative control. the results presented similar pattern to observed in FA-treated cells.

Next, we aimed to validate the functional activity of PGC-1α and Sirt 1 proteins in lipid metabolism in our cellular model by overexpressing them. This study was conducted considering that the results suggested a strict cellular fine-tuning in the Sirt1- PGC-1α axis when cells were transfected with the miR-1914-5p inhibitor.

**Fig 6:**
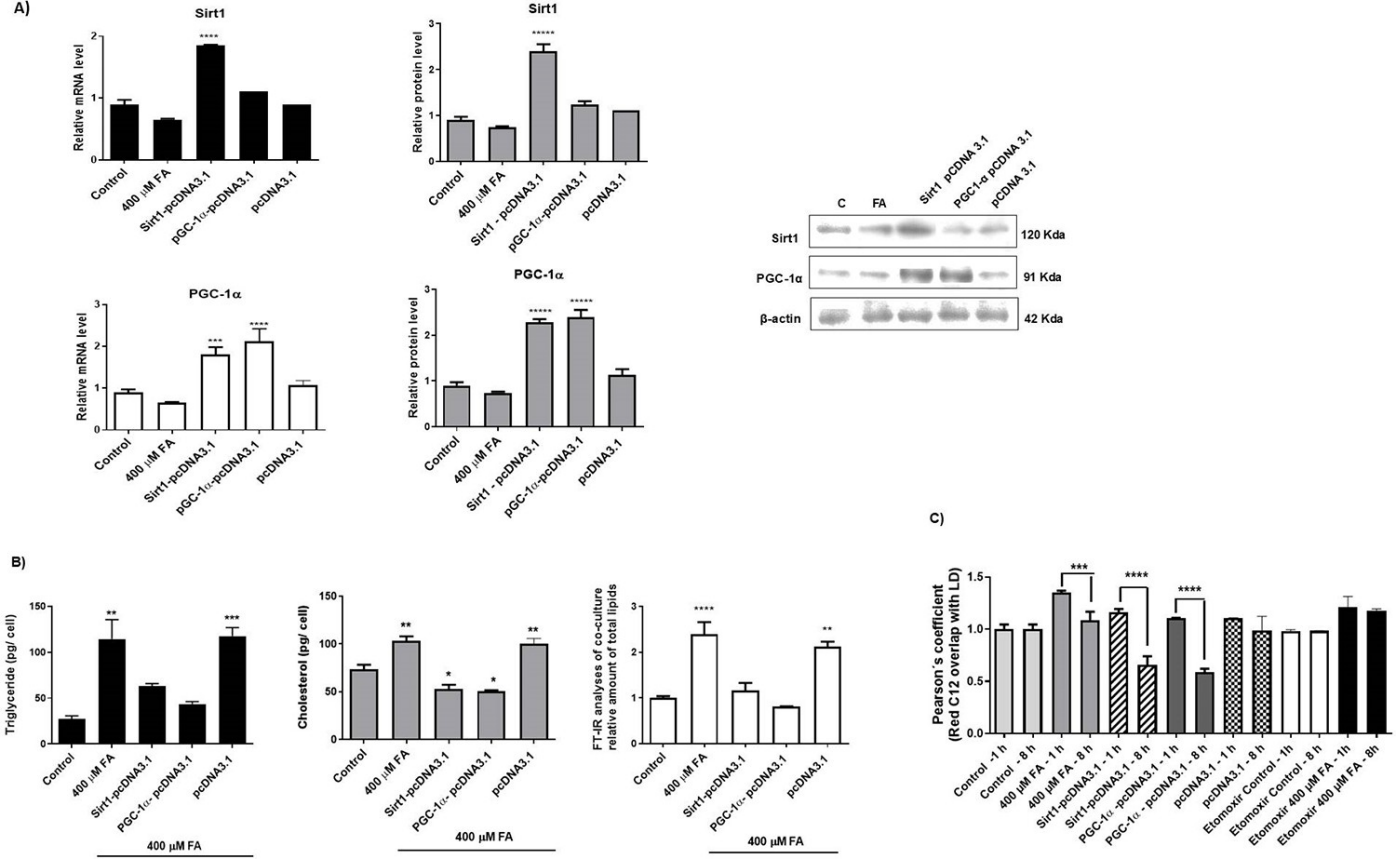
Overexpression of Sirt1 and PGC-1α in co-culture of hepatic cells to validate their functional activity on lipid metabolism in a *in vitro* cellular model of steatosis. A) Sirt1 and PGC-1α mRNA and protein levels in different groups of hepatic co-cultivated cells. B) Biochemical analyses of molecules correlated with lipid metabolism in different groups of cells. C) Beta-oxidation analyses to validate the effect of Sirt1 and PGC-1α overexpression in hepatic cells. The graphs and images present mean values of the average from at least three independent experiments. ANOVA testing showed significant differences (*p< 0.05) in the assays.

Cells in co-culture overexpressing Sirt1 (Sirt1-pcDNA3.1) or PGC-1α (PGC-1α-pcDNA3.1) (Fig 6A) reduced levels of TG, cholesterol, and total lipids (Fig 6B), when cultivated in culture media containing 400 μM of FA mixture, similar to the results observed in miR-inhibitor transfected cells. Both clones, Sirt1-pcDNA3.1 and PGC-1α-pcDNA3.1 overexpressed PGC-1α. This overexpression is observed in Sirt1-pcDNA3.1 clone, because Sirt1 indirect effects PGC-1α expression through FOXO1, as observed in miR-1914-5p inhibitor-transfected cells. Moreover, decreased levels of lipid droplets calculated by Pearson’s coefficient and the presence of Bodipy-C_12_ were observed in co-cultures overexpressing Sirt1 or PGC-1α (Fig 6C), suggesting increased beta-oxidation levels. The results combined corroborated the functional activity of the Sirt1- PGC-1α axis in miR-1914-5p-inhibitor-transfected cell in controlling the *in vitro* steatotic environment in hepatic cells.

### The systemic effects of the miR-1914-5p reinforce its modulatory effect on the *in vitro* steatotic cellular environment

Considering all the results in this study and the presence of potential miR-1914-5p target sequences on several miRNAs correlated to lipid and energetic metabolism metabolism (S2 Table), physical interaction between the miR-1914-5p and the 3’- UTR of the ACC2, ChREBP, PGC-1α, PPAR-γ, and Sirt1 mRNAs were evaluated in dual-luciferase assays. S5 Fig presents the results, which corroborated the physical interaction between the miRNA 1914-5p and each independent pGL3-construct containing the potential target sites of miRNA. Mutation on seed sequence disrupted the mRNA: miRNA interaction. When the miR-1914-5p inhibitor was used in the co-transfection, the luciferase production increased. For the analyses, only the LX-2 cell line was used, considering its high transfection efficiency [53] and to avoid some inappropriate luciferase background from the other cell used in the co-culture. Scramble miRNAs (mirVana™miRNAs, Nc) were also used as a negative control, and they did not cause relevant changes in the luciferase production, compared to the control culture.

The results supported the observation of increased mRNA levels of ACC2, ChREBP, PGC-1α, PPAR-γ, and Sirt1 in the miR-inhibitor group of cells (Figs 3–5) acts systemically in cells modulating lipid metabolism and the *in vitro* steatotic cellular environment, reinforcing the Sirt1-PGC-1α axis, in inducing steatotic markers of the metabolism. This observation can be considered as a valuable tool for the development of an innovative therapeutic approach to NAFLD treatment.

## Discussion

MAFLD and fatty liver are a worldwide health concern, and no effective therapy is currently available. In this study, we established an *in vitro* cellular model of steatosis to investigate mechanistic details of the effects of steatotic agents or chemical compounds in liver cell physiology. Due to the international policy of reducing the use of animal models in research, cell culture is considered an important tool in predictive toxicology analyses, and our model can contribute in this field.

In this study, we also investigated the molecular function of miR-1914-5p in controlling cellular lipid metabolism in a co-culture of HepG2 and LX-2 hepatic cells. The cells combined synergize their metabolites and cellular signaling, which mimics the steatotic environment [55]. In our analyses we demonstrated that the inhibition of the miR-1914-5p significantly reduced lipid levels in an *in vitro* steatotic environment by reducing TG, cholesterol, and total lipid levels (Fig 1). The miR-inhibitor also changed cellular metabolism and reduced the levels of oleic and palmitic fatty acids inside the cells; despite the fact that they were cultivated under high fatty conditions. Especially, the level of palmitic acid is reduced inside the cells in miR-inhibitor-transfected cells, compared to the levels found in the control cultures (Fig 2). The results suggested that this fatty acid was consumed/ or oxidated by cellular metabolism to avoid its intoxication, considering that palmitic acid is toxic and contributes to the establishment of steatotic environment [32,54]. This hypothesis is reinforced by the combined analyses of gene expression of molecules correlated with the energetic metabolism and oxidative processes. Fig 3 demonstrated an increased levels of gene expression of ACC2, PPARγ in miR-inhibitor transfected cells. The presence of an oxidative environment is reinforced by the analyses of the β-oxidation of FA in fluorescent pulse-chase analyses and PGC-1α levels, a master regulator of lipid oxidation [55] (Fig 5). In addition, the activation of alternative bioenergetic routes, such as the activation of the carbohydrate metabolism, suggested by the high expression levels of ChREBP in miR-inhibitor-transfected cells compared to the untransfected culture, reinforced an interconnected metabolism, which maintain cells alive. The homeostasis in miR-inhibitor-transfected cells is assured by a non-pro-inflammatory environment (S2 Fig) supported by low ROS production and a balanced mitochondrial membrane potential (S3 Fig).

Considering the energetic metabolism as an intricate molecular pathway, Sirt1 levels were investigated in miR-inhibitor transfected cells. This protein, deacetylates FOXO1, stimulating the expression and the activation PGC-1α, which modulates energetic metabolism, including down regulation of glucose metabolism (S4 Fig). In miR- inhibitor-transfected cells it was observed an increased transcriptional and protein levels of Sirt1, which- reinforced its close connection in regulating central elements correlated with lipid and energetic metabolism in those transfected cells. To mechanistic validate the function of Sirt1 and PGC-1α in controlling the molecular markers of a steatotic environment in our cellular model, the recombinant overexpression of those proteins demonstrated to control energetic metabolites inside the cells activating the lipid oxidation processes (Fig 6).

**Fig 7:**
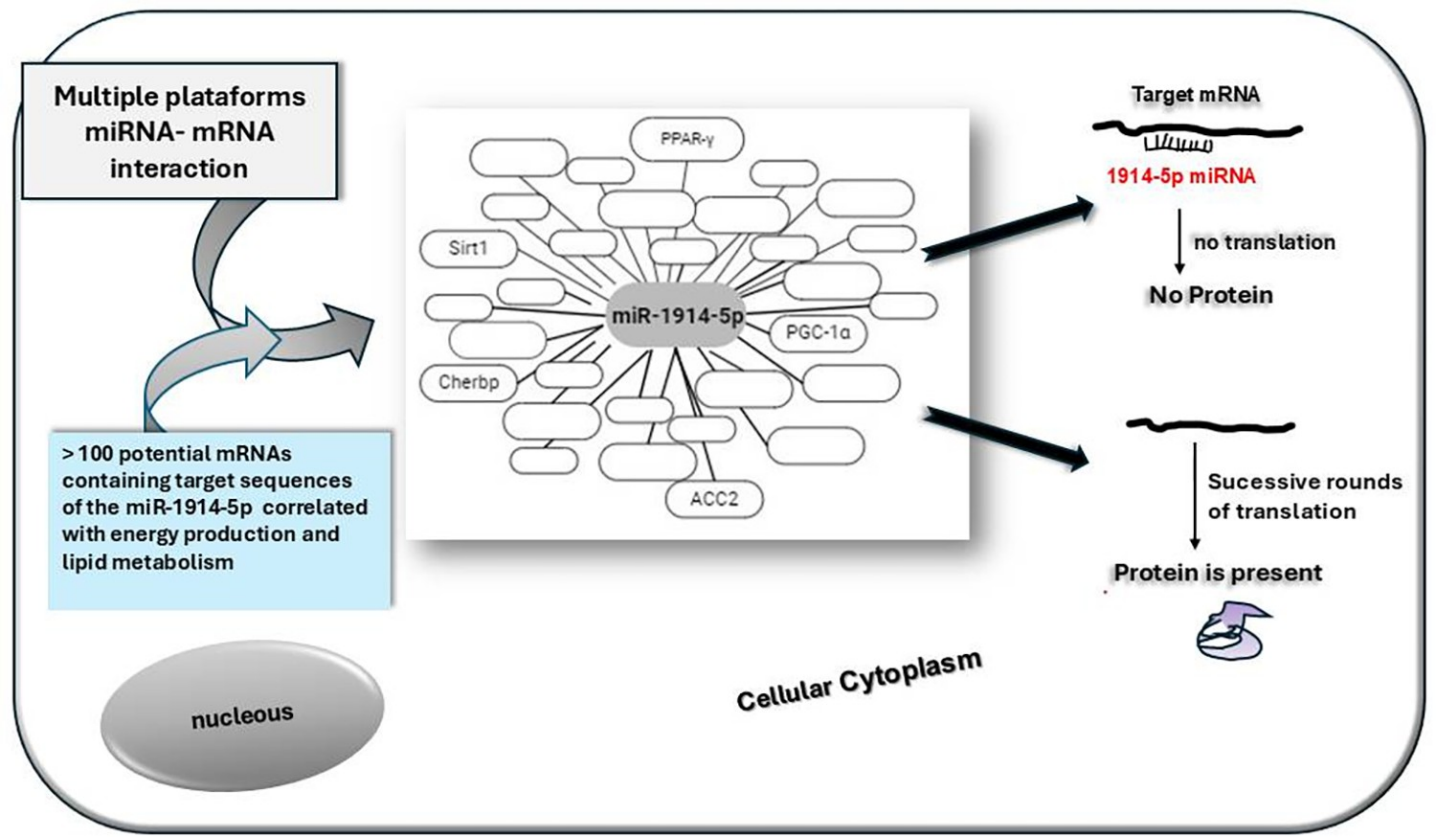
Schematic representation of multisystemic effects of miR-1914-5p in bioenergetic metabolism in co-culture of hepatic cells.

Moreover, considering the bioinformatic analyses that pointed to several potential mRNA target sequences to the miR-1914-5p (S2 Table), we evaluated physical interactions between miRNA:mRNA among some of them in a classical dual-luciferase reporter assay (S5 Fig). This assay reinforced the suggestion that the miR-1914-5p controls lipid and energetic metabolisms systemically, together with the Sit1-PGC-1α molecular routes (Fig 7).

In conclusion, the results demonstrated that the inhibition of miR-1914-5p contributed to the control of energetic metabolism, reducing lipid metabolites and even glucose levels in an *in vitro* cellular model of steatosis. Besides, the presence of potential target sequences to the miR-1914-5p in several mRNAs correlated to the production of energetic metabolites, and its physical intereaction with some of them, strongly suggest a multisystemic effects of the investigated miR in controlling energetic metabolism. However, the lack of orthologs of the miRNA to further validate its functional activity, should be observed, and translational approach using alternative models of investigation must be conducted before its preclinical investigation.

## Supporting information

S1 Table Oligonucleotides sequences used in qRT-PCR analyses. (DOCX)

S2 Table miRNA 1914-5p targets correlated with lipids and energetic metabolism. (DOCX)

S1 Fig Establishing *in vitro* steatotic cellular model. A) Different groups of cells (HepG2, LX2 and co-culture of HepG2 and LX-2 at the proportion rate of 7:3) were seeded in 96-wells plates and cultivated for 24 h under regular conditions. Next, different volumes of DMSO (v/v equivalent of FA) were added to the cultures. After 24 h cell viability was measured by MTT analyses; B) Triglycerides and cholesterol levels measurement after FA mixture addition to the cellular co-culture model D) Triglycerides and cholesterol levels for all the investigated conditions in isolated group of cells. The graphs represent the mean values of at least three independent experiments (*p<0.05).
(TIF)

S2 Fig Pro-inflammatory elements markers in different cellular groups. A) Annexin 1 mRNA levels in qRT-PCR analyses; B) Gas chromatography/ mass spectrometry (GC/MS) analyses of arachidonic acid (aa) in investigated group of cells. Graphs represent the mean values of at least three independent experiments (*p<0.05).
(TIF)

S3 Fig Mitochondrial activity. A) ROS production and B) mitochondrial membrane potential ((ΔΨm) production in HepG2: LX-2 (7:3) co-cultures in different groups of cells. Graphs represent the mean values of at least three independent experiments (*p<0.05).
(TIF)

S4 Fig Molecular elements of glucose metabolism A) Glucose levels and B) mRNA levels of G6Pase and PEPCK in in different groups of co-cultivated hepatic cells. Graphs represent the mean values of at least three independent experiments (*p<0.05).
(TIF)

S5 Fig Dual-luciferase assays of molecular connectors of energetic metabolism in hepatic cells. The analyses corroborated the physical interaction between the miRNA 1914-5p mimic and the 3’- UTR of ACC2, ChREBP, PGC-1α, PPAR-γ, and Sirt1. Representative target sequences of each independent 3’- UTR gene sequence or its mut-3’- UTR and the miR-1914-5p mimics or inhibitor or. Scramble miRNAs (mirVana™miRNAs were co-transfected in LX-2 cells to measure the luciferase activity. The graphs represent the mean values of at least three independent experiments (*p<0.05).
(TIF)

S1 Raw images Western blotting raw images.kcmm. (PDF)
