# Supplementary material for: Inhibition of the miR-1914-5p increases the oxidative metabolism in cellular model of steatosis by modulating the Sirt1-PGC-1α pathway and systemic cellular activity
Source: PLoS One. 2024 Nov 8;19(11):e0313185. doi: 10.1371/journal.pone.0313185 (PMC11548759; doi:10.1371/journal.pone.0313185)
Supplement: S1 Table — (DOCX) [file pone.0313185.s001.docx]

| **Gene** | **Oligonucleotide Forward (5’-3’)** | **Oligonucleotide Reverse (5’-3’)** |
| --- | --- | --- |
| β-ACTIN | CGGGACCTGACTGACTAC | CTCCTTAATGTCACGCAC |
| ACC1 | TGTAAGAGCTCATTTTGGAGGA | GAATCGAGAGTGCTGGTTCAG |
| ACC2 | GCAGCTGATGACCAACTTCA | TCCGGGTAGACTCACGAGAT |
| acly | CATCCGGAGGTAGATGTGCT | CGGATCTGGGCATAGTTCAT |
| acsl4 | ATGGATGATTGCAGCACAGA | CTGCTTCTTTGCCAAGTGTG |
| acsl5 | GAGCCCACTCCTGATGATGT | GGCTCCACAGCTGTACACAA |
| agpat1 | AATGGCTGGATCCTCTTCCT | GAGCAGCATTAGACGCAAGA |
| agpat2 | GTTCGTGCGAAGCTTCAAGT | TGGTTGGAGACGATGACACA |
| agpat3 | GCAGTCATCATCCTCAACCA | TTCTTAGCGAGGACCTTGGA |
| agpat4 | TGACTTTCTGTGTGGCTGGA | CCGATAATTGGGACATAGGC |
| ALDO | AGCCTGAGATCCTCCCTGAT | TTGTAGACAGCAGCCAGCAC |
| cd36 | GCAAAATCCACAGGAAGTGA | CGTCCTGGGTTACATTTTCC |
| chrebp | TGCAAACAGCTCTTCTCCAG | CGGACAAAAAGCAATTGAGG |
| CPT1A | GCTCATGGTGAACAGCAACT | TAAAGCAGGATGGCATGGAT |
| CPT2 | CCAGCCAGCTACCACTGACT | TTAGCAGCTGTGATGCCAGT |
| cs | CCATCCACAGTGACCATGAG | GCTGCAAAGGACAGGTAAGG |
| DGAT1 | GCCTTCTTCCACGAGTACCT | AGTGGGATCTGAGCCATCA |
| DGAT2 | TGAGTCTCTGAGCTCCATGC | AACCAGGTCAGCTCCATGAC |
| ENO | GATGATGCTGTGCCTTGATG | GCAGAGGAGAGCATTTCAGG |
| FASN | TCCTGCTGACCAAGAAGTCC | TCCTGCTGACCAAGAAGTCC |
| FOXO1 | GCCTGACCCAAGTGAAGAC | GCCCATTCTGCCATAGCC |
| GADPH | GATCATCAGCAATGCCTCCT | TGTGGTCATGAGTCCTTCCA |
| gpat1 | TCGATGAACACCAGATGGA | CAAGAATTGCTGCTGTCGAA |
| gpat3 | CAGAAGGGAGGCATTTGTGT | ATGAACCTGGCCAACCATAG |
| gpat4 | TACCTGCTGCGAATGATGAC | TATTCGCAAACTGGACAGCA |
| GPI | GAGACCATCACGAATGCAGA | AGACAGGGCAACAAAGTGCT |
| HK1 | AGGAATTTGACCTGGACGTG | CCCAACAATGAGTCCAACCT |
| hsl | TTTGAGATGCCACTGACTGC | GAGATGAGCCTGACGAGGAC |
| lpin1 | ATGAATTACGTGGGGCAGTT | ATGATGTCAATGCACCCTGA |
| lpin2 | TGAGCTCCGATGATGACAAG | TGATCCCCAGAATGGAAGAG |
| lpin3 | TTCAGCCTGACACAGAGGAT | GAGCTCTGAGTCTTGCTTTCCT |
| lxra | TCCACTACAATGTTCTGAGC | ACGCATGTAGGTGTCCAT |
| magl | GATCGCCTATGTGACAGCAA | GCTCCTTGTGGAGAACATGG |
| MLYCD | GACATCTCCAGCAACATCCA | CTGGGTCAAGCTGATGGAAT |
| PFK | CGATGATTCCATTTGTGTGC | CCTGTGCTCAAAATCCGTTT |
| PGAM | CCGACTGGTATTCCCATTGT | TCTTCATCCCCCAGAAACTG |
| PGK1 | CGAGCCAGCCAAAATAGAAG | CTCTGTGAGCAGTGCCAAAA |
| pgc1-α | CCAAAGGATGCGCTCTCGTT | CGGTGTCTGTAGTGGCTTGA |
| PKM | CGTGGATGATGGGCTTATTT | AAGGTTCACACCCTTCTTGC |
| PPARα | GGCCTCAGGCTATCATTACG | ACCAGCTTGAGTCGAATCGT |
| PPARγ | GGCTTCATGACAAGGGAGTTTC | AACTCAAACTTGGGCTCCATAAAG |
| rxr α | GTTCGCTAAGCTCTTGCTC | CATAAGGAAGGTGTCAATGG |
| rxrβ | CTGCCGCTATCAGAAGTGC | ATCCCCATCCTTGTCCTTTC |
| scd1 | GGACGATATCTCTAGCTCCT | TCGTCTCCAACTTATCTCCT |
| SIRT1 | GTTTTTGCCACCAAATCGTT | TCTGGCATGTCCCACTATCA |
| SREBP1c | AATCCGCCGCGCCTTGACAG | AAGTGCAATCCATGGCTCC |
| TPI | CAGACAAAGGTCATCGCAGA | TTGCAGTCTTGCCAGTACCA |
