# Supplementary material for: Inhibition of the miR-1914-5p increases the oxidative metabolism in cellular model of steatosis by modulating the Sirt1-PGC-1α pathway and systemic cellular activity
Source: PLoS One. 2024 Nov 8;19(11):e0313185. doi: 10.1371/journal.pone.0313185 (PMC11548759; doi:10.1371/journal.pone.0313185)
Supplement: S2 Table — (DOCX) [file pone.0313185.s002.docx]

| **Gene Symbol** | **Representative transcripts** | **Gene** | **Potential miRNA binding sites** |
| --- | --- | --- | --- |
| PAQR8 | ENST00000442253.2 | progestin and adipoQ receptor family member VIII | 2 |
| AWAT1 | ENST00000374521.3 | acyl-CoA wax alcohol acyltransferase 1 | 1 |
| ACADSB | ENST00000358776.4 | acyl-CoA dehydrogenase, short/branched chain | 3 |
| HSD11B2 | ENST00000326152.5 | hydroxysteroid (11-beta) dehydrogenase 2 | 2 |
| INPP5A | ENST00000368594.3 | inositol polyphosphate-5-phosphatase, 40kDa | 2 |
| LSS | ENST00000397728.3 | lanosterol synthase (2,3-oxidosqualene-lanosterol cyclase) | 4 |
| CYB5D1 | ENST00000571846.1 | cytochrome b5 domain containing 1 | 1 |
| MRPS27 | ENST00000261413.5 | mitochondrial ribosomal protein S27 | 3 |
| GSK3B | ENST00000264235.8 | glycogen synthase kinase 3 beta | 1 |
| BHMT | ENST00000274353.5 | betaine--homocysteine S-methyltransferase | 1 |
| HSDL1 | ENST00000219439.4 | hydroxysteroid dehydrogenase like 1 | 1 |
| LHFPL2 | ENST00000515007.2 | lipoma HMGIC fusion partner-like 2 | 2 |
| SCD | ENST00000370355.2 | stearoyl-CoA desaturase (delta-9-desaturase) | 1 |
| HILPDA | ENST00000257696.4 | hypoxia inducible lipid droplet-associated | 1 |
| GLP2R | ENST00000262441.5 | glucagon-like peptide 2 receptor | 1 |
| PIK3C2G | ENST00000433979.1 | phosphatidylinositol-4-phosphate 3-kinase, catalytic subunit type 2 gamma | 1 |
| GSTM5 | ENST00000369813.1 | glutathione S-transferase mu 5 | 2 |
| CS | ENST00000548567.1 | citrate synthase | 1 |
| GSTZ1 | ENST00000216465.5 | glutathione S-transferase zeta 1 | 2 |
| ACAD11 | ENST00000355458.3 | acyl-CoA dehydrogenase family, member 11 | 2 |
| PGAM1 | ENST00000334828.5 | phosphoglycerate mutase 1 (brain) | 1 |
| SAP30L | ENST00000297109.6 | SAP30-like | 1 |
| AOAH | ENST00000538464.1 | acyloxyacyl hydrolase (neutrophil) | 1 |
| ALDH1A2 | ENST00000249750.4 | aldehyde dehydrogenase 1 family, member A2 | 1 |
| PGAM5 | ENST00000498926.2 | phosphoglycerate mutase family member 5 | 2 |
| PGAM4 | ENST00000458128.1 | phosphoglycerate mutase family member 4 | 1 |
| ELOVL7 | ENST00000508821.1 | ELOVL fatty acid elongase 7 | 1 |
| PITPNB | ENST00000335272.5 | phosphatidylinositol transfer protein, beta | 1 |
| IPMK | ENST00000373935.3 | inositol polyphosphate multikinase | 1 |
| CYP8B1 | ENST00000316161.4 | cytochrome P450, family 8, subfamily B, polypeptide 1 | 2 |
| ACACA | ENST00000353139.5 | acetyl-CoA carboxylase alpha | 2 |
| MLYCD | ENST00000262430.4 | malonyl-CoA decarboxylase | 1 |
| PI4KA | ENST00000255882.6 | phosphatidylinositol 4-kinase, catalytic, alpha | 1 |
| RAI14 | ENST00000265109.3 | retinoic acid induced 14 | 1 |
| LMF2 | ENST00000380796.3 | lipase maturation factor 2 | 1 |
| MINOS1 | ENST00000322753.6 | mitochondrial inner membrane organizing system 1 | 2 |
| INPP5B | ENST00000373027.1 | inositol polyphosphate-5-phosphatase, 75kDa | 1 |
| PLCXD1 | ENST00000381657.2 | phosphatidylinositol-specific phospholipase C, X domain containing 1 | 1 |
| LDLRAP1 | ENST00000374338.4 | low density lipoprotein receptor adaptor protein 1 | 1 |
| DUOXA1 | ENST00000558996.1 | dual oxidase maturation factor 1 | 1 |
| CPT1B | ENST00000360719.2 | carnitine palmitoyltransferase 1B (muscle) | 1 |
| COQ9 | ENST00000262507.6 | coenzyme Q9 homolog (S. cerevisiae) | 1 |
| RDH8 | ENST00000591589.1 | retinol dehydrogenase 8 (all-trans) | 1 |
| NOX5 | ENST00000260364.5 | NADPH oxidase, EF-hand calcium binding domain 5 | 2 |
| PLA2G2F | ENST00000375102.3 | phospholipase A2, group IIF | 3 |
| ACSS1 | ENST00000323482.4 | acyl-CoA synthetase short-chain family member 1 | 1 |
| GPIHBP1 | ENST00000330824.2 | glycosylphosphatidylinositol anchored high density lipoprotein binding protein 1 | 1 |
| OXR1 | ENST00000312046.6 | oxidation resistance 1 | 1 |
| EHHADH | ENST00000231887.3 | enoyl-CoA, hydratase/3-hydroxyacyl CoA dehydrogenase | 1 |
| IGFBP3 | ENST00000381086.5 | insulin-like growth factor binding protein 3 | 1 |
| NDUFAF7 | ENST00000002125.4 | NADH dehydrogenase (ubiquinone) complex I, assembly factor 7 | 1 |
| CPT1C | ENST00000323446.5 | carnitine palmitoyltransferase 1C | 1 |
| NDOR1 | ENST00000427047.2 | NADPH dependent diflavin oxidoreductase 1 | 2 |
| CYP46A1 | ENST00000261835.3 | cytochrome P450, family 46, subfamily A, polypeptide 1 | 1 |
| PIK3R6 | ENST00000311434.9 | phosphoinositide-3-kinase, regulatory subunit 6 | 1 |
| PDK2 | ENST00000503176.1 | pyruvate dehydrogenase kinase, isozyme 2 | 1 |
| CYP1A1 | ENST00000379727.3 | cytochrome P450, family 1, subfamily A, polypeptide 1 | 1 |
| FNTB | ENST00000246166.2 | farnesyltransferase, CAAX box, beta | 2 |
| NAT16 | ENST00000300303.2 | N-acetyltransferase 16 (GCN5-related, putative) | 1 |
| SLN | ENST00000531293.1 | sarcolipin | 1 |
| GTDC1 | ENST00000392869.2 | glycosyltransferase-like domain containing 1 | 2 |
| COASY | ENST00000420359.1 | CoA synthase | 1 |
| ACSBG1 | ENST00000258873.4 | acyl-CoA synthetase bubblegum family member 1 | 2 |
| PLCD3 | ENST00000322765.5 | phospholipase C, delta 3 | 1 |
| ITPRIPL2 | ENST00000381440.3 | inositol 1,4,5-trisphosphate receptor interacting protein-like 2 | 1 |
| PLA2G15 | ENST00000566188.1 | phospholipase A2, group XV | 2 |
| CYP2S1 | ENST00000310054.4 | cytochrome P450, family 2, subfamily S, polypeptide 1 | 1 |
| DEGS1 | ENST00000323699.4 | delta(4)-desaturase, sphingolipid 1 | 1 |
| CYP27C1 | ENST00000335247.7 | cytochrome P450, family 27, subfamily C, polypeptide 1 | 1 |
| FFAR2 | ENST00000599180.2 | free fatty acid receptor 2 | 1 |
| PREX1 | ENST00000396220.1 | phosphatidylinositol-3,4,5-trisphosphate-dependent Rac exchange factor 1 | 2 |
| PIGU | ENST00000452740.2 | phosphatidylinositol glycan anchor biosynthesis, class U | 1 |
| STARD8 | ENST00000252336.6 | StAR-related lipid transfer (START) domain containing 8 | 1 |
| APOL2 | ENST00000358502.5 | apolipoprotein L, 2 | 1 |
| ENOSF1 | ENST00000383578.3 | enolase superfamily member 1 | 2 |
| OSBPL11 | ENST00000296220.5 | oxysterol binding protein-like 11 | 1 |
| PLCG1 | ENST00000244007.3 | phospholipase C, gamma 1 | 3 |
| PLCE1 | ENST00000371380.3 | phospholipase C, epsilon 1 | 1 |
| NDUFA4L2 | ENST00000556732.1 | NADH dehydrogenase (ubiquinone) 1 alpha subcomplex, 4-like 2 | 1 |
| MLXIP | ENST00000319080.7 | MLX interacting protein | 2 |
| OGDH | ENST00000222673.5 | oxoglutarate (alpha-ketoglutarate) dehydrogenase (lipoamide) | 1 |
| LRP5L | ENST00000402859.2 | low density lipoprotein receptor-related protein 5-like | 1 |
| DLST | ENST00000334220.4 | dihydrolipoamide S-succinyltransferase (E2 component of 2-oxo-glutarate complex) | 1 |
| IP6K3 | ENST00000451316.1 | inositol hexakisphosphate kinase 3 | 1 |
| LHFPL5 | ENST00000360215.1 | lipoma HMGIC fusion partner-like 5 | 1 |
| PLA2G7 | ENST00000274793.7 | phospholipase A2, group VII (platelet-activating factor acetylhydrolase, plasma) | 1 |
| ACSF3 | ENST00000317447.4 | acyl-CoA synthetase family member 3 | 1 |
| DGKG | ENST00000265022.3 | diacylglycerol kinase, gamma 90kDa | 1 |
| LRP8 | ENST00000306052.6 | low density lipoprotein receptor-related protein 8, apolipoprotein e receptor | 3 |
| LDLRAD2 | ENST00000344642.2 | low density lipoprotein receptor class A domain containing 2 | 2 |
| DGKD | ENST00000264057.2 | diacylglycerol kinase, delta 130kDa | 1 |
| ITPK1 | ENST00000267615.6 | inositol-tetrakisphosphate 1-kinase | 1 |
| ITPR2 | ENST00000381340.3 | inositol 1,4,5-trisphosphate receptor, type 2 | 2 |
| PTGES | ENST00000340607.4 | prostaglandin E synthase | 1 |
| PDE3B | ENST00000282096.4 | phosphodiesterase 3B, cGMP-inhibited | 1 |
| ADH1B | ENST00000305046.8 | alcohol dehydrogenase 1B (class I), beta polypeptide | 1 |
| TFPI | ENST00000392365.1 | tissue factor pathway inhibitor (lipoprotein-associated coagulation inhibitor) | 1 |
| PDE2A | ENST00000334456.5 | phosphodiesterase 2A, cGMP-stimulated | 1 |
| ACOT9 | ENST00000379303.5 | acyl-CoA thioesterase 9 | 2 |
| IDH1 | ENST00000345146.2 | isocitrate dehydrogenase 1 (NADP+), soluble | 1 |
| DBT | ENST00000370132.4 | dihydrolipoamide branched chain transacylase E2 | 2 |
| PIGL | ENST00000225609.5 | phosphatidylinositol glycan anchor biosynthesis, class L | 2 |
| APOOL | ENST00000373173.2 | apolipoprotein O-like | 1 |
| PLP1 | ENST00000303958.2 | proteolipid protein 1 | 1 |
| CYB5R3 | ENST00000361740.4 | cytochrome b5 reductase 3 | 2 |
| PLD5 | ENST00000536534.2 | phospholipase D family, member 5 | 1 |
| HSD17B13 | ENST00000302219.6 | hydroxysteroid (17-beta) dehydrogenase 13 | 1 |
| CYP4F11 | ENST00000326742.8 | cytochrome P450, family 4, subfamily F, polypeptide 11 | 1 |
| INPP4A | ENST00000409016.4 | inositol polyphosphate-4-phosphatase, type I, 107kDa | 2 |
| HHAT | ENST00000413764.2 | hedgehog acyltransferase | 1 |
| LRAT | ENST00000336356.3 | lecithin retinol acyltransferase (phosphatidylcholine--retinol O-acyltransferase) | 1 |
| FASN | ENST00000306749.2 | fatty acid synthase | 1 |
| PFKFB4 | ENST00000232375.3 | 6-phosphofructo-2-kinase/fructose-2,6-biphosphatase 4 | 1 |
| IDH3G | ENST00000370093.1 | isocitrate dehydrogenase 3 (NAD+) gamma | 1 |
| HAGHL | ENST00000549114.1 | hydroxyacylglutathione hydrolase-like | 1 |
| PLCH2 | ENST00000449969.1 | phospholipase C, eta 2 | 4 |
| ACBD5 | ENST00000396271.3 | acyl-CoA binding domain containing 5 | 2 |
| LPIN1 | ENST00000256720.2 | lipin 1 | 1 |
| MGLL | ENST00000434178.2 | monoglyceride lipase | 2 |
| OSBPL3 | ENST00000313367.2 | oxysterol binding protein-like 3 | 1 |
| STARD3 | ENST00000336308.5 | StAR-related lipid transfer (START) domain containing 3 | 1 |
| HSD17B11 | ENST00000358290.4 | hydroxysteroid (17-beta) dehydrogenase 11 | 1 |
| AGPAT6 | ENST00000396987.3 | 1-acylglycerol-3-phosphate O-acyltransferase 6 | 1 |
| IGF2R | ENST00000356956.1 | insulin-like growth factor 2 receptor | 2 |
| STS | ENST00000217961.4 | steroid sulfatase (microsomal), isozyme S | 1 |
| AGPAT4 | ENST00000366911.5 | 1-acylglycerol-3-phosphate O-acyltransferase 4 | 3 |
| FOXN1 | ENST00000226247.2 | forkhead box N1 | 1 |
| SIRT3 | ENST00000382743.4 | sirtuin 3 | 1 |
| APOL3 | ENST00000397293.2 | apolipoprotein L, 3 | 1 |
| RAI1 | ENST00000353383.1 | retinoic acid induced 1 | 1 |
| FA2H | ENST00000219368.3 | fatty acid 2-hydroxylase | 1 |
| GLYAT | ENST00000344743.3 | glycine-N-acyltransferase | 1 |
| LPIN2 | ENST00000261596.4 | lipin 2 | 1 |
| HDLBP | ENST00000391975.1 | high density lipoprotein binding protein | 3 |
| RXRB | ENST00000374685.4 | retinoid X receptor, beta | 1 |
| CYP27B1 | ENST00000228606.4 | cytochrome P450, family 27, subfamily B, polypeptide 1 | 1 |
| CYP11B1 | ENST00000292427.4 | cytochrome P450, family 11, subfamily B, polypeptide 1 | 1 |
| GPD1 | ENST00000301149.3 | glycerol-3-phosphate dehydrogenase 1 (soluble) | 1 |
| PIP4K2B | ENST00000269554.3 | phosphatidylinositol-5-phosphate 4-kinase, type II, beta | 2 |
| LHFPL4 | ENST00000287585.6 | lipoma HMGIC fusion partner-like 4 | 4 |
| NDUFA5 | ENST00000471770.1 | NADH dehydrogenase (ubiquinone) 1 alpha subcomplex, 5 | 1 |
| PLCXD3 | ENST00000377801.3 | phosphatidylinositol-specific phospholipase C, X domain containing 3 | 1 |
| SREBF2 | ENST00000361204.4 | sterol regulatory element binding transcription factor 2 | 1 |
| SORD | ENST00000267814.9 | sorbitol dehydrogenase | 1 |
| PITPNA | ENST00000313486.7 | phosphatidylinositol transfer protein, alpha | 1 |
| OARD1 | ENST00000373154.2 | O-acyl-ADP-ribose deacylase 1 | 1 |
| PNLIPRP3 | ENST00000369230.3 | pancreatic lipase-related protein 3 | 1 |
| H6PD | ENST00000377403.2 | hexose-6-phosphate dehydrogenase (glucose 1-dehydrogenase) | 1 |
| PTGFRN | ENST00000393203.2 | prostaglandin F2 receptor inhibitor | 1 |
| LPGAT1 | ENST00000366997.4 | lysophosphatidylglycerol acyltransferase 1 | 1 |
| PTGER3 | ENST00000370924.4 | prostaglandin E receptor 3 (subtype EP3) | 1 |
| STARD5 | ENST00000302824.6 | StAR-related lipid transfer (START) domain containing 5 | 1 |
| RDH13 | ENST00000415061.3 | retinol dehydrogenase 13 (all-trans/9-cis) | 2 |
| PC | ENST00000529047.1 | pyruvate carboxylase | 1 |
| DAGLA | ENST00000257215.5 | diacylglycerol lipase, alpha | 1 |
| SUCNR1 | ENST00000362032.5 | succinate receptor 1 | 1 |
| SDHAF2 | ENST00000543265.1 | succinate dehydrogenase complex assembly factor 2 | 1 |
| RETSAT | ENST00000295802.4 | retinol saturase (all-trans-retinol 13,14-reductase) | 2 |
| MRPS11 | ENST00000325844.4 | mitochondrial ribosomal protein S11 | 2 |
| SPTLC2 | ENST00000216484.2 | serine palmitoyltransferase, long chain base subunit 2 | 2 |
| CYP11B2 | ENST00000323110.2 | cytochrome P450, family 11, subfamily B, polypeptide 2 | 1 |
| MRPS16 | ENST00000372945.3 | mitochondrial ribosomal protein S16 | 1 |
| PIP5K1C | ENST00000335312.3 | phosphatidylinositol-4-phosphate 5-kinase, type I, gamma | 2 |
| TIMM22 | ENST00000327158.4 | translocase of inner mitochondrial membrane 22 homolog (yeast) | 1 |
| CRTC1 | ENST00000338797.6 | CREB regulated transcription coactivator 1 | 1 |
| GSR | ENST00000221130.5 | glutathione reductase | 4 |
| PIK3CA | ENST00000263967.3 | phosphatidylinositol-4,5-bisphosphate 3-kinase, catalytic subunit alpha | 1 |
| INPP5K | ENST00000421807.2 | inositol polyphosphate-5-phosphatase K | 1 |
| FADS6 | ENST00000310226.6 | fatty acid desaturase 6 | 1 |
| PPARGC1B | ENST00000309241.5 | peroxisome proliferator-activated receptor gamma, coactivator 1 beta | 1 |
| IPPK | ENST00000287996.3 | inositol 1,3,4,5,6-pentakisphosphate 2-kinase | 1 |
| INSR | ENST00000341500.5 | insulin receptor | 1 |
| GPLD1 | ENST00000230036.1 | glycosylphosphatidylinositol specific phospholipase D1 | 3 |
| FADS1 | ENST00000350997.7 | fatty acid desaturase 1 | 2 |
| RXRA | ENST00000481739.1 | retinoid X receptor, alpha | 1 |
| MECR | ENST00000373791.3 | mitochondrial trans-2-enoyl-CoA reductase | 1 |
| NR1H3 | ENST00000467728.1 | nuclear receptor subfamily 1, group H, member 3 | 1 |
| SREBF1 | ENST00000395757.1 | sterol regulatory element binding transcription factor 1 | 1 |
| DHCR24 | ENST00000371269.3 | 24-dehydrocholesterol reductase | 1 |
| UCP3 | ENST00000314032.4 | uncoupling protein 3 (mitochondrial, proton carrier) | 1 |
| PDE11A | ENST00000286063.6 | phosphodiesterase 11A | 1 |
| SLC27A1 | ENST00000252595.7 | solute carrier family 27 (fatty acid transporter), member 1 | 1 |
| LPP | ENST00000312675.4 | LIM domain containing preferred translocation partner in lipoma | 1 |
| PPARA | ENST00000396000.2 | peroxisome proliferator-activated receptor alpha | 1 |
| GPCPD1 | ENST00000379019.4 | glycerophosphocholine phosphodiesterase GDE1 homolog (S. cerevisiae) | 1 |
| CLYBL | ENST00000376355.3 | citrate lyase beta like | 3 |
| IGF1R | ENST00000268035.6 | insulin-like growth factor 1 receptor | 1 |
| MBOAT2 | ENST00000305997.3 | membrane bound O-acyltransferase domain containing 2 | 2 |
| LDHD | ENST00000300051.4 | lactate dehydrogenase D | 1 |
| MRPS25 | ENST00000253686.2 | mitochondrial ribosomal protein S25 | 1 |
| CYP51A1 | ENST00000003100.8 | cytochrome P450, family 51, subfamily A, polypeptide 1 | 1 |
| DGKH | ENST00000261491.5 | diacylglycerol kinase, eta | 1 |
| IGF2BP1 | ENST00000290341.3 | insulin-like growth factor 2 mRNA binding protein 1 | 1 |
| OXNAD1 | ENST00000285083.5 | oxidoreductase NAD-binding domain containing 1 | 1 |
| CDS2 | ENST00000460006.1 | CDP-diacylglycerol synthase (phosphatidate cytidylyltransferase) 2 | 3 |
| PDK3 | ENST00000441463.2 | pyruvate dehydrogenase kinase, isozyme 3 | 1 |
| LRP10 | ENST00000359591.4 | low density lipoprotein receptor-related protein 10 | 1 |
| NDUFB9 | ENST00000276689.3 | NADH dehydrogenase (ubiquinone) 1 beta subcomplex, 9, 22kDa | 1 |
| GSTO1 | ENST00000539281.1 | glutathione S-transferase omega 1 | 1 |
| IRS4 | ENST00000372129.2 | insulin receptor substrate 4 | 1 |
| LDLRAD4 | ENST00000399848.3 | low density lipoprotein receptor class A domain containing 4 | 1 |
| PIK3C3 | ENST00000262039.4 | phosphatidylinositol 3-kinase, catalytic subunit type 3 | 2 |
| SDHAF1 | ENST00000378887.2 | succinate dehydrogenase complex assembly factor 1 | 1 |
| GK5 | ENST00000392993.2 | glycerol kinase 5 (putative) | 1 |
| PGK1 | ENST00000373316.4 | phosphoglycerate kinase 1 | 1 |
| INSL4 | ENST00000239316.4 | insulin-like 4 (placenta) | 1 |
| AMACR | ENST00000335606.6 | alpha-methylacyl-CoA racemase | 1 |
| FTO | ENST00000471389.1 | fat mass and obesity associated | 4 |
| CMC1 | ENST00000466830.1 | COX assembly mitochondrial protein 1 homolog (S. cerevisiae) | 2 |
| AGK | ENST00000355413.4 | acylglycerol kinase | 1 |
| VLDLR | ENST00000382100.3 | very low density lipoprotein receptor | 1 |
| PLA2G16 | ENST00000323646.5 | phospholipase A2, group XVI | 1 |
| COX6B1 | ENST00000246554.3 | cytochrome c oxidase subunit VIb polypeptide 1 (ubiquitous) | 2* |
| LRPAP1 | ENST00000500728.2 | low density lipoprotein receptor-related protein associated protein 1 | 2 |
| DPM1 | ENST00000371588.5 | dolichyl-phosphate mannosyltransferase polypeptide 1, catalytic subunit | 1 |
| DGAT1 | ENST00000332324.4 | diacylglycerol O-acyltransferase 1 | 1 |
| LMF1 | ENST00000262301.11 | lipase maturation factor 1 | 1 |
| BPIFA3 | ENST00000375454.3 | BPI fold containing family A, member 3 | 1 |
| PIGP | ENST00000360525.4 | phosphatidylinositol glycan anchor biosynthesis, class P | 2 |
| LDHA | ENST00000227157.4 | lactate dehydrogenase A | 1 |
| NDUFAF4 | ENST00000316149.7 | NADH dehydrogenase (ubiquinone) complex I, assembly factor 4 | 1 |
| ACSF2 | ENST00000427954.2 | acyl-CoA synthetase family member 2 | 1 |
| PDK1 | ENST00000282077.3 | pyruvate dehydrogenase kinase, isozyme 1 | 1 |
| FAR2 | ENST00000536681.3 | fatty acyl CoA reductase 2 | 1 |
| LDLRAD1 | ENST00000371362.3 | low density lipoprotein receptor class A domain containing 1 | 1 |
| G6PC2 | ENST00000421979.1 | glucose-6-phosphatase, catalytic, 2 | 1 |
| PTGDR | ENST00000553372.1 | prostaglandin D2 receptor (DP) | 1 |
| PEX7 | ENST00000541292.1 | peroxisomal biogenesis factor 7 | 1 |
| FAAH | ENST00000243167.8 | fatty acid amide hydrolase | 2 |
| FFAR1 | ENST00000246553.2 | free fatty acid receptor 1 | 1 |
| HSD11B1 | ENST00000367028.2 | hydroxysteroid (11-beta) dehydrogenase 1 | 1 |
| NQO2 | ENST00000338130.2 | NAD(P)H dehydrogenase, quinone 2 | 1 |
| LIPG | ENST00000261292.4 | lipase, endothelial | 1 |
| RARRES3 | ENST00000255688.3 | retinoic acid receptor responder (tazarotene induced) 3 | 1 |
| ACSM2B | ENST00000329697.6 | acyl-CoA synthetase medium-chain family member 2B | 1 |
| PIK3CG | ENST00000359195.3 | phosphatidylinositol-4,5-bisphosphate 3-kinase, catalytic subunit gamma | 1 |
| APOB | ENST00000233242.1 | apolipoprotein B | 1 |
| ACSM2A | ENST00000573854.1 | acyl-CoA synthetase medium-chain family member 2A | 1 |
| CYP20A1 | ENST00000356079.4 | cytochrome P450, family 20, subfamily A, polypeptide 1 | 2* |
| APOLD1 | ENST00000356591.4 | apolipoprotein L domain containing 1 | 1 |
| G6PC | ENST00000253801.2 | glucose-6-phosphatase, catalytic subunit | 1 |
