# Supplementary figures and images for: Inhibition of the miR-1914-5p increases the oxidative metabolism in cellular model of steatosis by modulating the Sirt1-PGC-1α pathway and systemic cellular activity
Source: PLoS One. 2024 Nov 8;19(11):e0313185. doi: 10.1371/journal.pone.0313185 (PMC11548759; doi:10.1371/journal.pone.0313185)

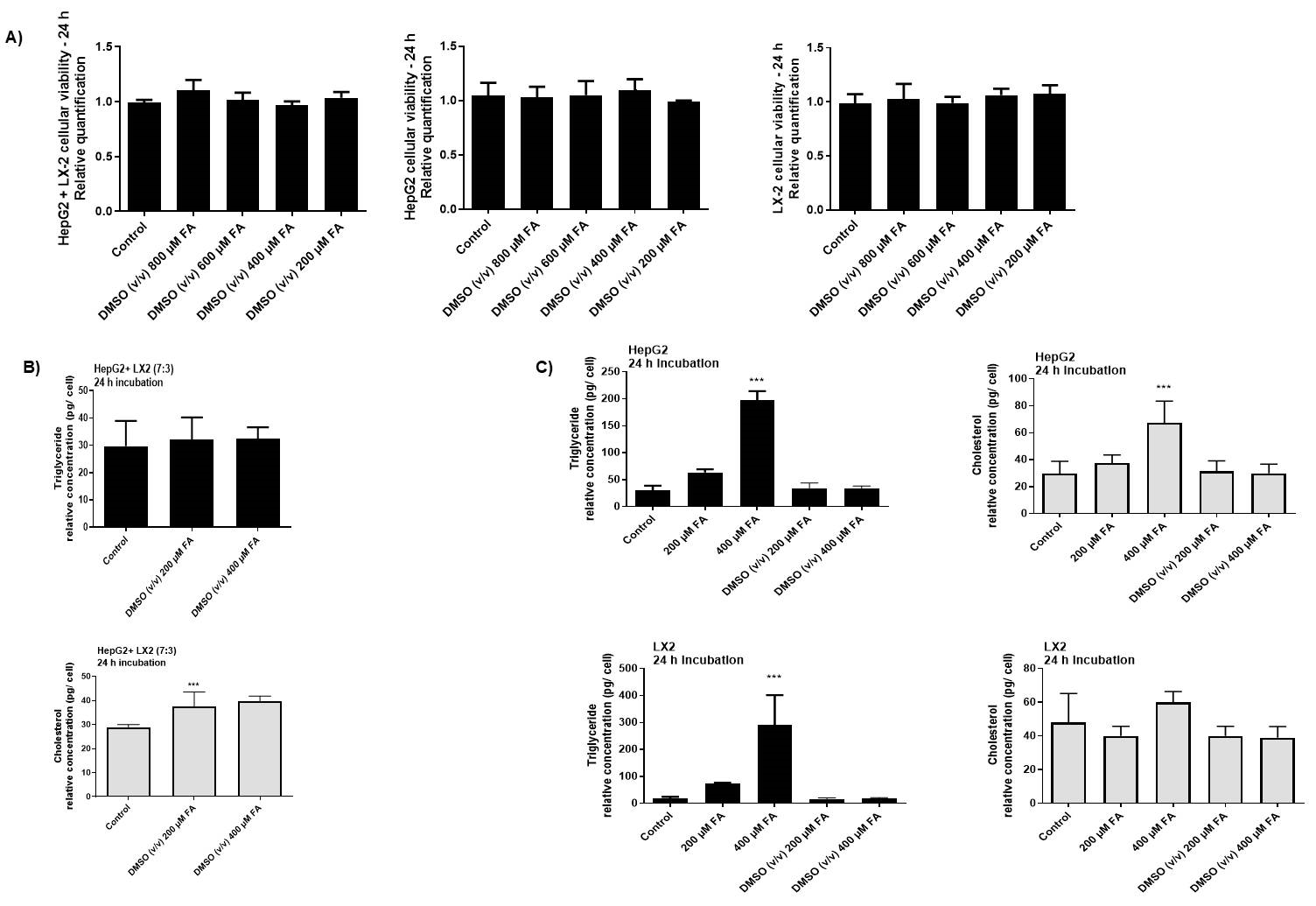

Supplement: S1 Fig — A) Different groups of cells (HepG2, LX2 and co-culture of HepG2 and LX-2 at the proportion rate of 7:3) were seeded in 96-wells plates and cultivated for 24 h under regular conditions. Next, different volumes of DMSO (v/v equivalent of FA) were added to the cultures. After 24 h cell viability was measured by MTT analyses; B) Triglycerides and cholesterol levels measurement after FA mixture addition to the cellular co-culture model D) Triglycerides and cholesterol levels for all the investigated conditions in isolated group of cells. The graphs represent the mean values of at least three independent experiments (*p<0.05). (TIF) [file pone.0313185.s003.tif]

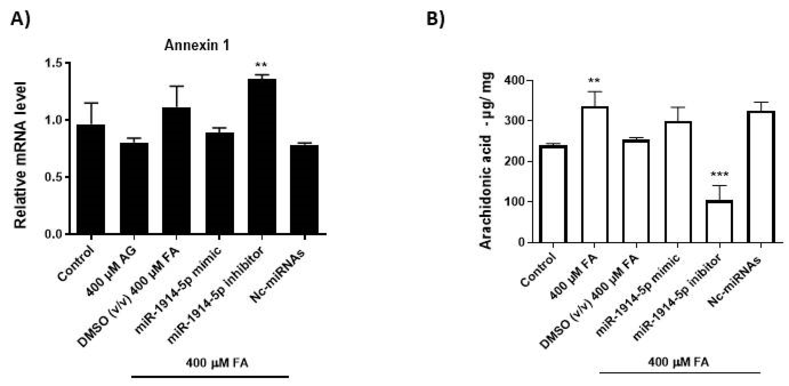

Supplement: S2 Fig — A) Annexin 1 mRNA levels in qRT-PCR analyses; B) Gas chromatography/ mass spectrometry (GC/MS) analyses of arachidonic acid (aa) in investigated group of cells. Graphs represent the mean values of at least three independent experiments (*p<0.05). (TIF) [file pone.0313185.s004.tif]

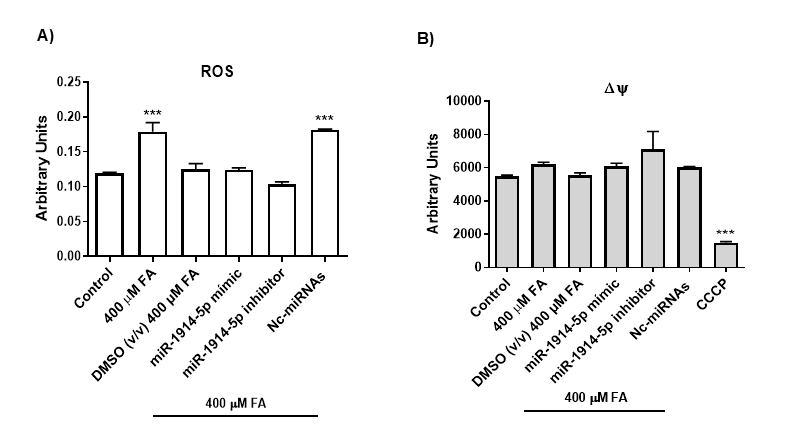

Supplement: S3 Fig — A) ROS production and B) mitochondrial membrane potential ((ΔΨm) production in HepG2: LX-2 (7:3) co-cultures in different groups of cells. Graphs represent the mean values of at least three independent experiments (*p<0.05). (TIF) [file pone.0313185.s005.tif]

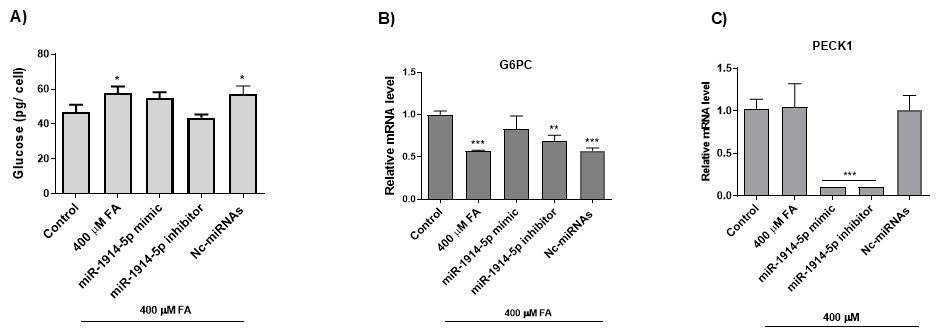

Supplement: S4 Fig — Molecular elements of glucose metabolism A) Glucose levels and B) mRNA levels of G6Pase and PEPCK in in different groups of co-cultivated hepatic cells. Graphs represent the mean values of at least three independent experiments (*p<0.05). (TIF) [file pone.0313185.s006.tif]

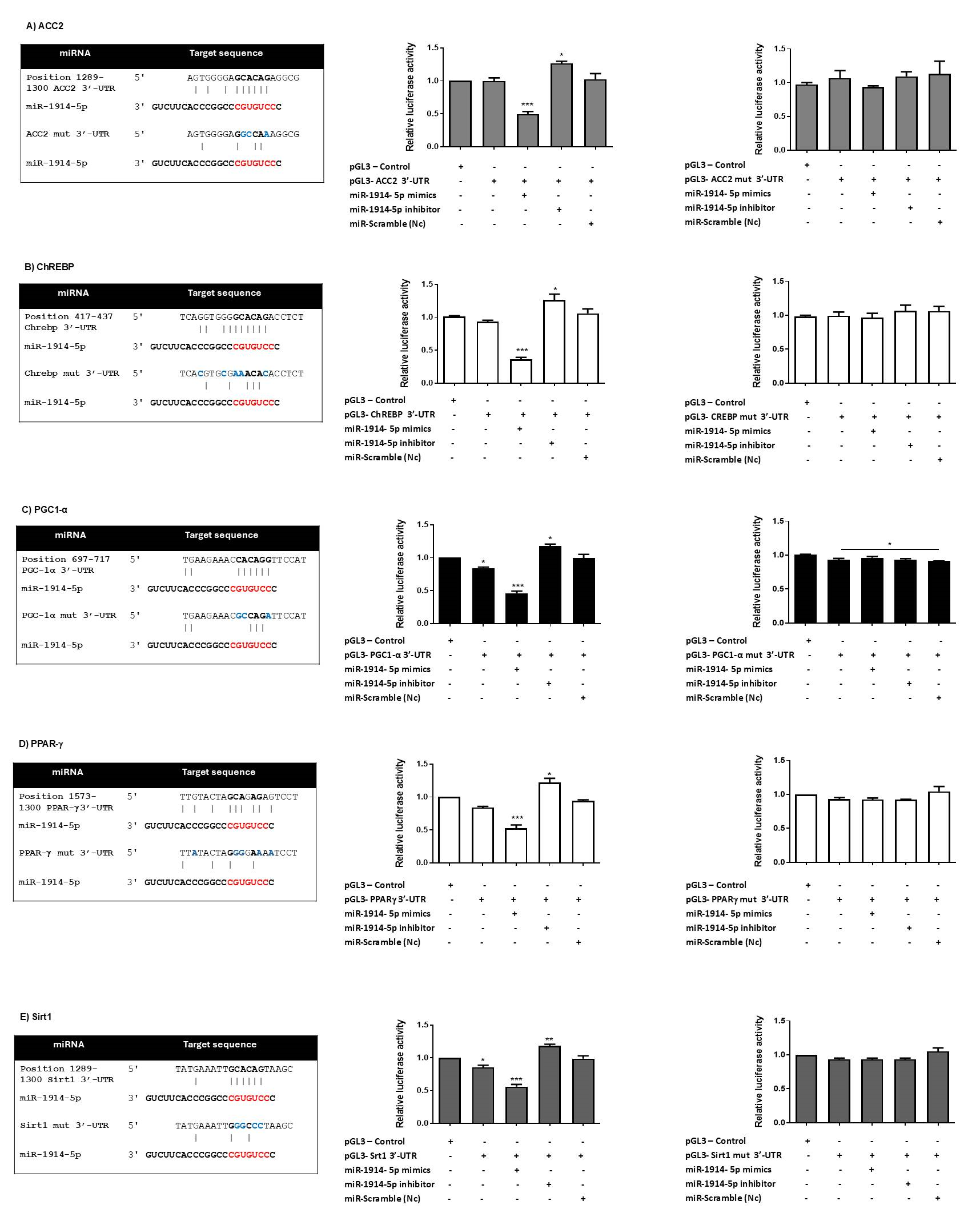

Supplement: S5 Fig — The analyses corroborated the physical interaction between the miRNA 1914-5p mimic and the 3’- UTR of ACC2, ChREBP, PGC-1α, PPAR-γ, and Sirt1. Representative target sequences of each independent 3’- UTR gene sequence or its mut-3’- UTR and the miR-1914-5p mimics or inhibitor or. Scramble miRNAs (mirVana™miRNAs were co-transfected in LX-2 cells to measure the luciferase activity. The graphs represent the mean values of at least three independent experiments (*p<0.05). (TIF) [file pone.0313185.s007.tif]
