# Supplementary material for: Inhibition of the miR-1914-5p increases the oxidative metabolism in cellular model of steatosis by modulating the Sirt1-PGC-1α pathway and systemic cellular activity
Source: PLoS One. 2024 Nov 8;19(11):e0313185. doi: 10.1371/journal.pone.0313185 (PMC11548759; doi:10.1371/journal.pone.0313185)
Supplement: S1 Raw images — (PDF) [file pone.0313185.s008.pdf]

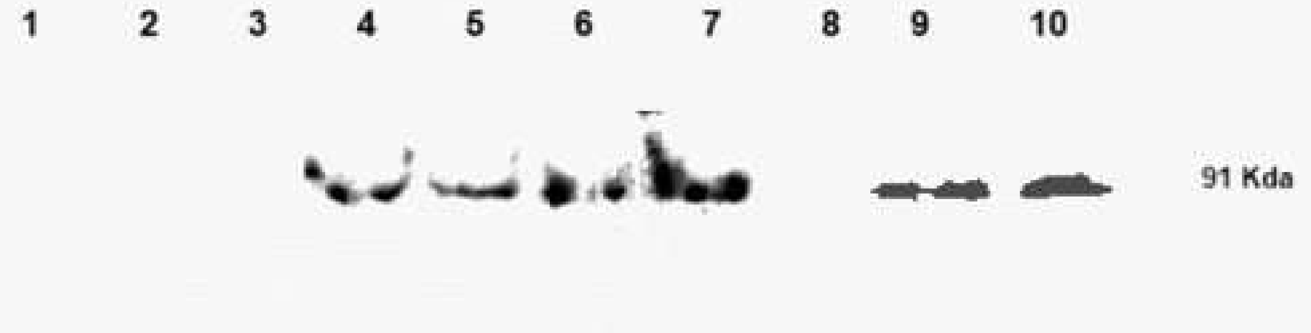

Imager Information
Imager Model C-DiGit
Imager Name LICOR-CDG-004381
Acquisition Times 12 minutes - Resolution 196 µm
Focus Offset Scan Area (cm) X: 0,0 Y: 0,0 W: 10,3
H: 10,2 Position
Source Information
Acquisition Software LI-COR Acquisition v2.2.0.99

1- empty lane
2 - empty lane
3 - empty lane
4 - control
5 - FA
6 – mimics + FA
7 – inhibitor + FA
8 - empty lane
9 - DMSO
10 – Negative Control (Nc)

1st ab- β-actin (1:800) – 16 h incubation
2nd ab- goat α -rabbit IgG HRP (1:6000) – 2 h incubation

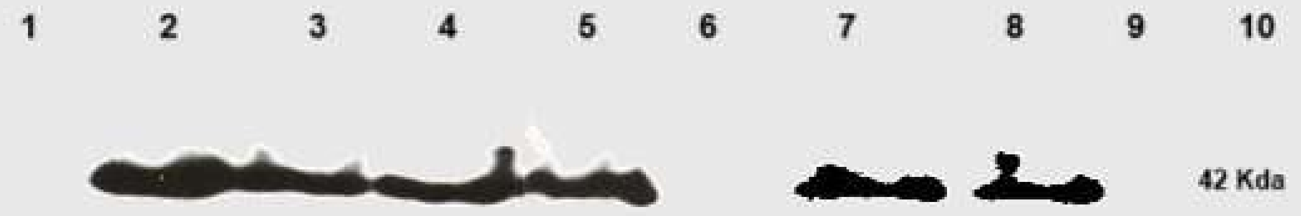

Imager Information
Imager Model C-DiGit
Imager Name LICOR-CDG-004381
Acquisition Times 12 minutes - Resolution 196 µm
Focus Offset Scan Area (cm) X: 0,0 Y: 0,0 W: 10,3
H: 10,2 Position
Source Information
Acquisition Software LI-COR Acquisition v2.2.0.99

1- empty lane
2 - control
3 - FA
4 -mimics + FA
5 - inhibitor + FA
6 – empty lane
7 - DMSO
8 - Negative Control (Nc)
9 - empty lane
10 - empty lane

1st ab- β-actin (1:800) –16 h incubation
2nd ab- goat α -rabbit IgG HRP (1:6000) – 2 h incubation

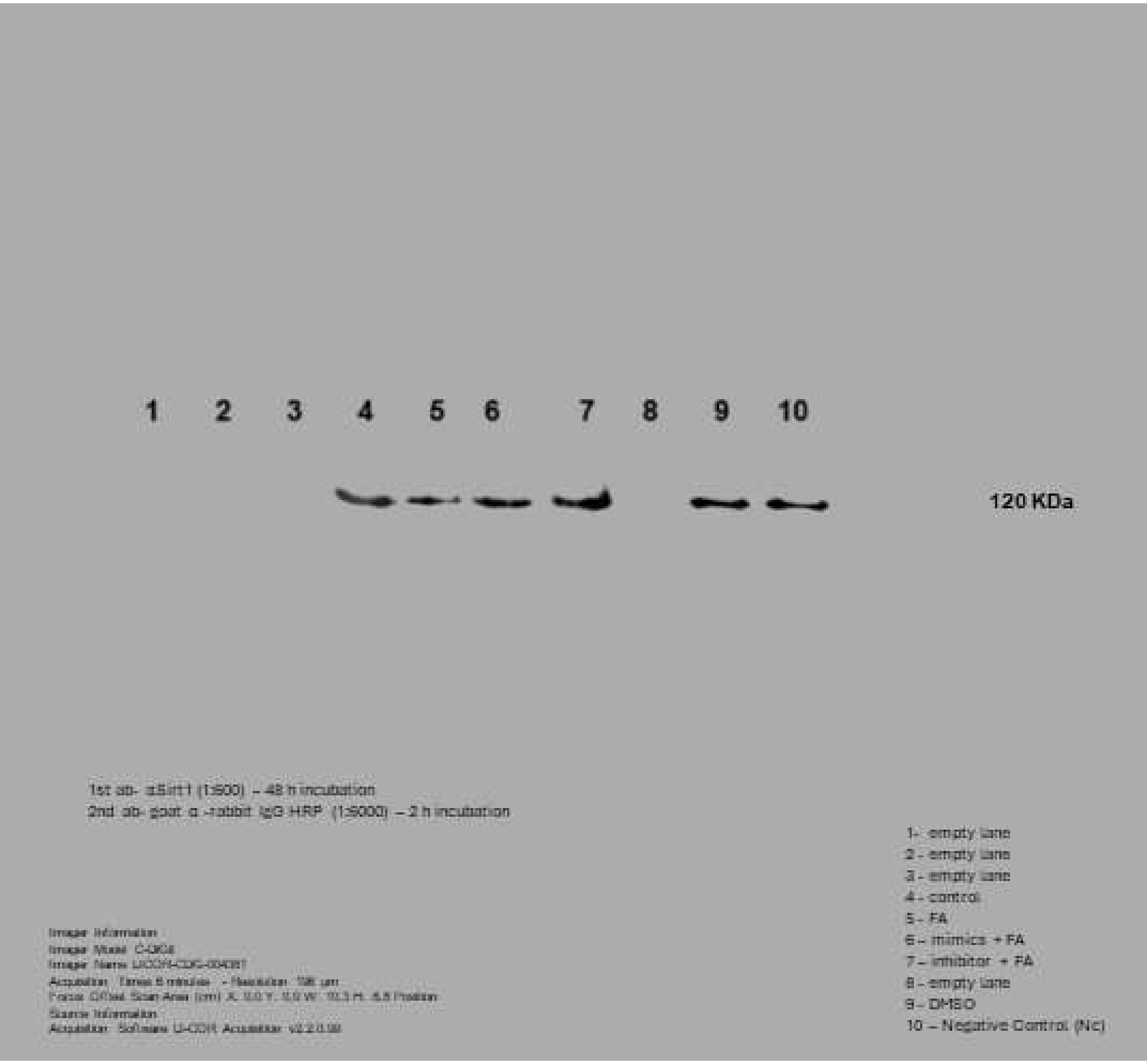
1 2 3 4 5 6 7 8 9 10
120 KDa
1st ab- αSirt1 (1:600) – 48 h incubation
2nd ab- goat α -rabbit IgG HRP (1:5000) – 2 h incubation
Image Information
Source Information
1- empty lane
2 - empty lane
3 - empty lane
4 - control
5 - FA
6 – mimics + FA
7 – inhibitor + FA
8 - empty lane
9 - DMSO
10 – Negative Control (Nc)

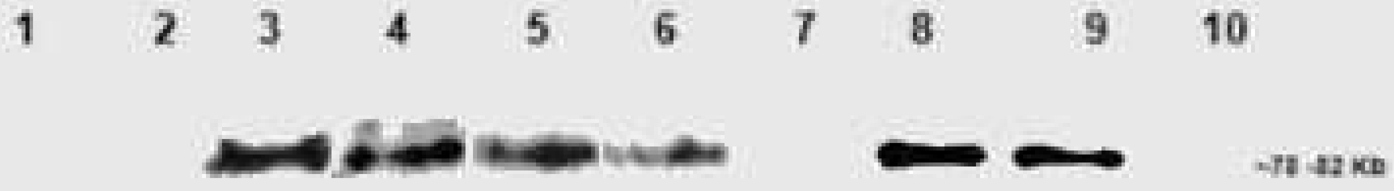

1- empty lane
2 - empty lane
3 - control
4 - FA
5 – mimics + FA
6 – inhibitor + FA
7 – empty lane
8 - DMSO
9 - Negative Control (Nc)
10 – empty lane

1st ab- α-acetFOXO-1 (1:500) – 48 h incubation
2nd ab- goat α -rabbit IgG HRP (1:5000) – 2 h incubation

Imager Information
Imager Model: C-DiGit
Imager Name: LICOR-CDG-[illegible]
Acquisition Time: 12 minutes – Resolution: [illegible] µm
Focus Offset Scan Area (cm) X: 0.0 Y: 0.0 W: 10.3 H: 14.2 Position
Source Information
Acquisition Software: LI-COR Acquisition v2.2.0.[illegible]

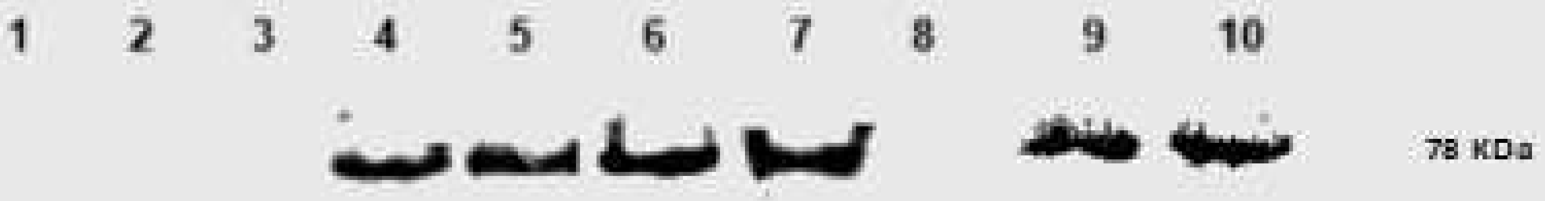

1st ab- αFOXO-1 (1:800) – 24 h incubation
2nd ab- goat α -rabbit IgG HRP (1:6000) – 2 h incubation

1- empty lane
2 - empty lane
3 - empty lane
4 - control
5 - FA
6 – mimics + FA
7 – inhibitor + FA
8 - empty lane
9 - DMSO
10 – Negative Control (Nc)

Image Information
Image Model C-DiGit
Image Name LICOR-CDG-[illegible]
Acquisition Time 12 minutes - Resolution [illegible] µm
Focus Offset Scan Area (cm) X: 0.0 Y: 0.0 W: 10.3 H: 10.2 Position
Source Information
Acquisition Software LI-COR Acquisition v2.2.0.[illegible]

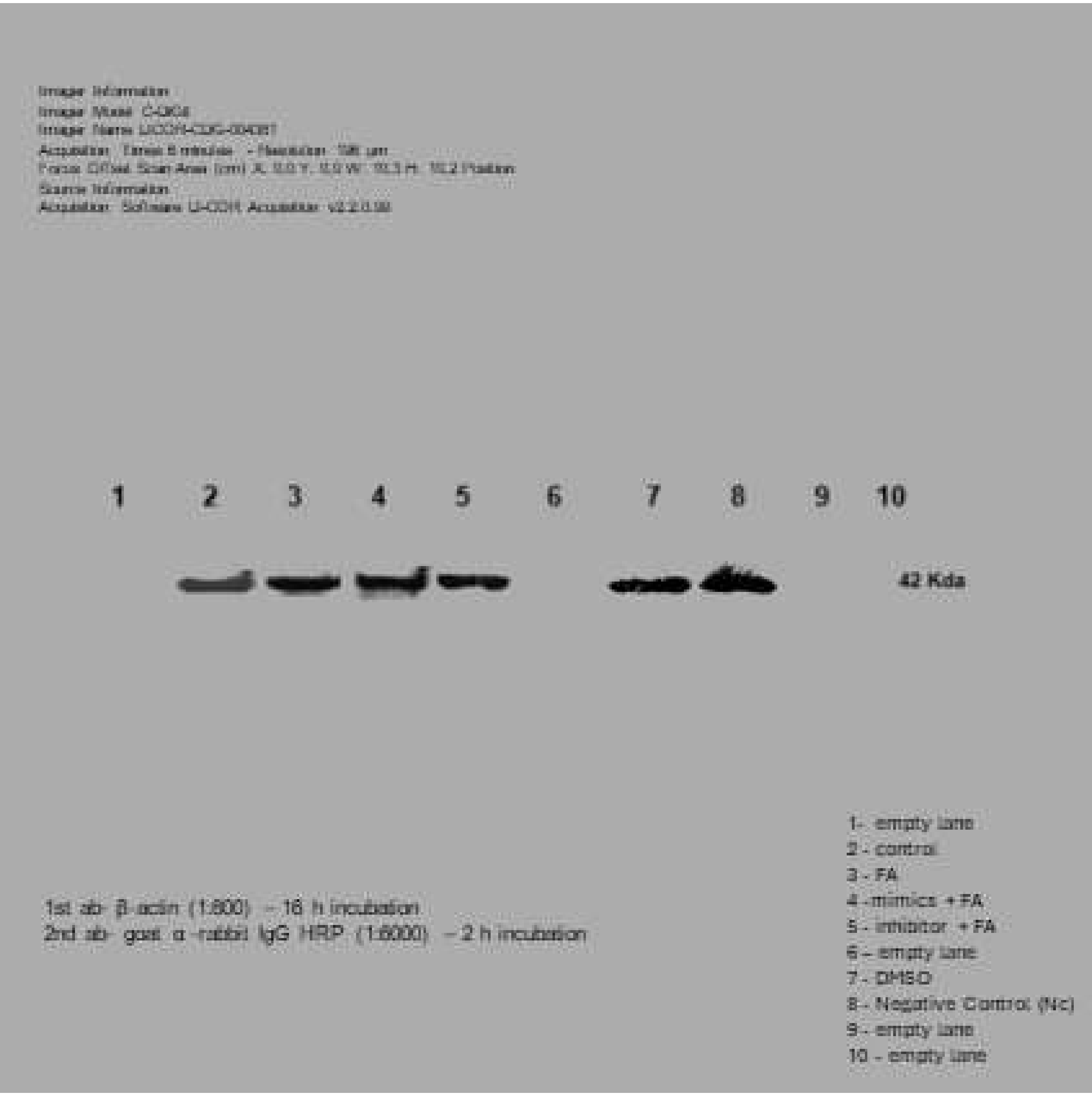
Image Information
Source Information
1
2
3
4
5
6
7
8
9
10
42 Kda
1st ab- β-actin (1:800) – 16 h incubation
2nd ab- goat α -rabbit IgG HRP (1:6000) – 2 h incubation
1- empty lane
2 - control
3 - FA
4 -mimics + FA
5 - inhibitor + FA
6 – empty lane
7 - DMSO
8 - Negative Control (Nc)
9 - empty lane
10 - empty lane

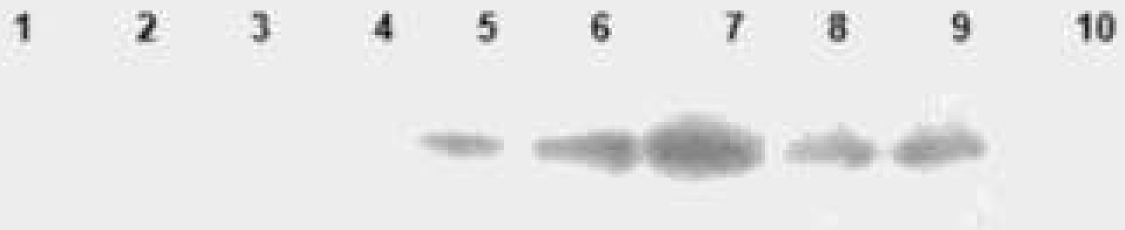

1st ab- αSirt1 (1:900) – 48 h incubation
2nd ab- goat α -rabbit IgG HRP (1:9000) – 2 h incubation

1- empty lane
2 - empty lane
3 - empty lane
4 - control
5 - FA
6 – Sirt1-pCDNA 3.1
7 – PGC1-α-pCDNA 3.1
8. -pCDNA 3.1
9 - empty lane
10 – empty lane

Imager Information
Imager Model C-DiGit
Imager Name LICOR-CDG-004381
Acquisition Times 12 minutes - Resolution 196 µm
Focus Offset Scan Area (cm) X: 0,0 Y: 0,0 W: 10,2
H: 9,32 Position
Source Information
Acquisition Software LI-COR Acquisition v2.2.0.99

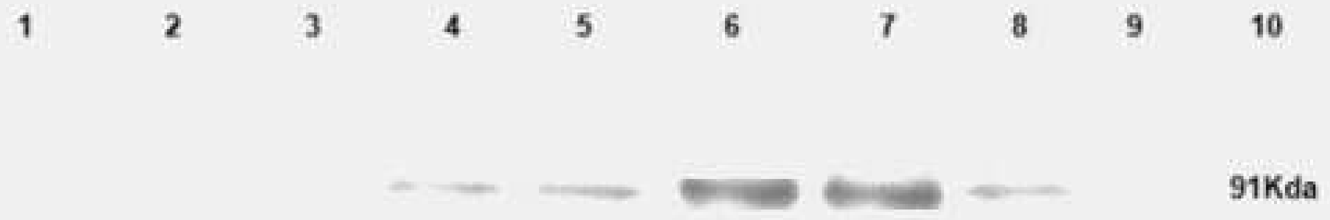

1- empty lane
2 - empty lane
3 – empty lane
4 - control
5 –FA
6 – Sirt1- pCDNA 3.1
7 – PGC1-α - pCDNA 3.1
8 - pCDNA 3.1
9 – empty lane
10 – empty lane

1st ab- PGC1-αn (1:400) – 48 h incubation
2nd ab- goat α-rabbit IgG HRP (1:8000) – 2 h incubation

Imager Information
Imager Model C-DiGit
Imager Name LICOR-CDG-004381
Acquisition Times 12 minutes - Resolution 196 µm
Focus Offset Scan Area (cm) X: 0,0 Y: 0,0 W: 9,3 H: 9,32 Position
Source Information
Acquisition Software LI-COR Acquisition v2.2.0.99

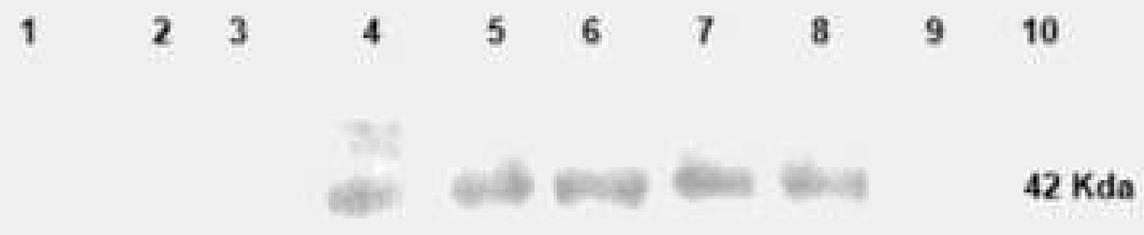

1- empty lane
2 - empty lane
3 - empty lane
4 - control
5 - FA
6 – Sirt1-pCDNA 3.1
7 – PGC1-α-pCDNA 3.1
8. -pCDNA 3.1
9 - empty lane
10 – empty lane

1st ab- β-actin (1:800) –16 h incubation
2nd ab- goat α -rabbit IgG HRP (1:9000) – 2 h incubation

Imager Information
Imager Model C-DiGit
Imager Name LICOR-CDG-004381
Acquisition Times 12 minutes - Resolution 196 µm
Focus Offset Scan Area (cm) X: 0,0 Y: 0,0 W: 9,3 H: 9,32 Position
Source Information
Acquisition Software LI-COR Acquisition v2.2.0.99
